# Effects of Pilates-Based Exercise on Mental Health, Psychological Well-Being, and Quality of Life: A Systematic Review and Meta-Analysis

**DOI:** 10.3390/sports14050171

**Published:** 2026-04-23

**Authors:** Ioannis Tsartsapakis, Aglaia Zafeiroudi, Charilaos Kouthouris

**Affiliations:** 1 Department of Physical Education & Sport Science at Serres, Aristotle University of Thessaloniki, 62100 Serres, Greece; 2 Department Physical Education & Sport Science, University of Thessaly, 42100 Trikala, Greece; kouthouris@uth.gr

**Keywords:** pilates, mental health, psychological well-being, quality of life, exercise intervention, remote training, meta-analysis, randomized trials

## Abstract

This systematic review and meta-analysis examined the impact of Pilates-based exercise on mental health, psychological well-being, and quality of life (QoL) across clinical and healthy populations. Thirty-two randomized and quasi-experimental trials (total *N* = 1264) were included, representing adolescents, adults, and older adults across diverse clinical and non-clinical groups. Outcomes encompassed depressive symptoms, anxiety, QoL, self-esteem, and well-being. The unadjusted random-effects model indicated a suggestive but statistically inconclusive overall effect (*p* = 0.061). However, adjusting for outcome type via meta-regression yielded a statistically significant pooled effect (*g* = 0.393, *p* = 0.023). Substantial heterogeneity remained across studies (*I*^2^ = 91.7%). Meta-regression identified outcome type as the only significant moderator, whereas age group, delivery mode, and clinical status did not significantly influence the pooled effect. Subgroup analyses suggested comparable benefits between remote and in-person delivery formats in general adult samples. Evidence from individual studies indicated that supervised, face-to-face instruction may be advantageous for older adults. Low-frequency programs, including once-weekly sessions, were also associated with improvements, although variability in intervention duration and structure limits conclusions regarding optimal dosage. Sensitivity analyses confirmed the stability of the pooled effect. Overall, the findings support Pilates as a feasible exercise modality with demonstrated benefits for positive psychosocial outcomes (QoL and self-esteem), while evidence for negative psychological indicators (e.g., depression, anxiety) remains limited or non-significant. Future research should standardize outcome measures, report training parameters consistently, and examine contextual factors contributing to heterogeneity in psychosocial responses.

## 1. Introduction

Promoting psychological well-being and managing mental health disorders have become central priorities in global public health. Across diverse demographic groups, from adolescents experiencing academic pressures [1] to older adults and clinical populations managing chronic pain [2], there is an increasing demand for safe, non-pharmacological interventions to address depression, anxiety, and diminished quality of life (QoL) [1,2]. Within this context, mind–body practices have gained prominence as therapeutic strategies that integrate physical functionality with psychological resilience. Unlike conventional aerobic or resistance-based exercise, mind–body interventions emphasize attentional focus, controlled breathing, and proprioceptive engagement, offering a holistic approach that is adaptable to a wide range of environments and functional capacities [3].

Originally developed as a system for physical rehabilitation, the Pilates method has evolved into a widely adopted mind–body practice. Pilates requires sustained internal focus, coordinated breathing, and precise motor control, distinguishing it from traditional exercise modalities [4,5]. A growing body of literature suggests that Pilates may confer benefits across multiple populations [6,7]. For example, it has been associated with improvements in postural stability and reductions in fear of falling among older adults [6], has been used as a complementary therapy for clinical groups such as breast cancer survivors [7], and has been linked to enhanced physical self-perception in healthy young adults [8]. Recent trials further illustrate the breadth of its applications, including improvements in QoL among individuals with Multiple Sclerosis [9], reductions in psychological distress in obese adolescents [10], and enhanced self-esteem among school teachers experiencing occupational stress [11]. These examples highlight the method’s relevance across both clinical and non-clinical populations.

Importantly, the psychological benefits of Pilates appear to be closely tied to its physical mechanisms. Improvements in body awareness and physical self-perception have been associated with enhanced self-esteem and reductions in social appearance anxiety [8,12]. For clinical populations, reductions in pain and improvements in functional mobility may serve as mediators of decreased depressive symptoms and improved QoL [13]. These findings suggest that Pilates may influence psychological outcomes through interconnected physical and cognitive pathways.

Conceptual models of exercise and mental health further propose that physical activity influences psychological outcomes through neurobiological, psychosocial (e.g., self-esteem, resilience), and behavioral pathways [14], offering a useful framework for interpreting the mechanisms through which mind–body interventions such as Pilates may exert their effects.

Despite its widespread use, the meta-analytical evidence on the psychosocial effects of Pilates remains limited and outdated. Earlier reviews, including Fleming and Herring [15], provided preliminary support for reductions in anxiety and depressive symptoms, but these analyses were constrained by small samples, narrow outcome scopes, and a predominant focus on negative psychological indicators. Importantly, they did not incorporate positive constructs such as QoL or self-esteem, nor did they evaluate the rapidly emerging digital delivery formats that have become central in the post-pandemic era [16]. As a result, the current literature lacks an integrated synthesis that reflects both the breadth of contemporary psychosocial outcomes and the diversity of modern Pilates interventions.

Moreover, the rapid expansion of digital health interventions, accelerated by the COVID-19 pandemic, has introduced new delivery formats that earlier meta-analyses could not evaluate. While recent trials suggest that online Pilates may be comparable to in-person instruction for healthy adults [17], other studies indicate that certain clinical populations, such as individuals with Long COVID, may benefit more from face-to-face supervision due to cognitive fatigue and attentional limitations [18].

A key objective of the present meta-analysis is to address these gaps by examining sources of heterogeneity through a structured meta-regression approach. Previous research has suggested that the efficacy of Pilates may vary according to intervention characteristics and participant profiles, yet these moderators have not been systematically synthesized. Specifically, this study investigates delivery mode (online versus face-to-face) to determine whether digital formats yield psychosocial outcomes comparable to traditional supervised instruction [17,18]. Additionally, clinical status and age are examined as potential moderators to assess whether the psychosocial effects of Pilates differ between healthy populations, where preventative health is emphasized, and clinical groups, where functional recovery and pain management are central [6,7,13]. By evaluating these moderators, the study aims to move beyond overall effect-size estimation and provide a more nuanced understanding of how Pilates interventions interact with individual characteristics.

To date, no comprehensive and updated meta-analysis has simultaneously evaluated both positive and negative psychosocial outcomes across the contemporary, post-pandemic Pilates literature. Therefore, the primary aim of this study is to quantify the overall effect of the Pilates method on psychosocial outcomes by synthesizing the most recent randomized and controlled trials. Secondarily, through meta-regression analyses, this study seeks to clarify the extent to which delivery mode, clinical status, and age contribute to heterogeneity, thereby providing evidence-based insights for healthcare professionals and researchers regarding the application of Pilates as a versatile mind–body intervention.

In this review, ‘psychosocial outcomes’ is utilized as an overarching umbrella term. This is further subdivided into ‘negative psychological indicators’ (e.g., depression, anxiety) and ‘positive well-being markers’ (e.g., quality of life, self-esteem).

## 2. Materials and Methods

This systematic review and meta-analysis was conducted in accordance with PRISMA 2020 [19] guidelines and was prospectively registered in PROSPERO (CRD420261333634). All methodological procedures followed the preregistered protocol without deviations.

### 2.1. Search Strategy

A comprehensive search was performed across PubMed, Scopus, Web of Science, PsycINFO, CINAHL, and CENTRAL from database inception until March 2026. The search strategy combined controlled vocabulary and free-text terms related to Pilates and psychosocial outcomes. Boolean operators, truncation, and database-specific filters were applied to maximize sensitivity. The Boolean string used in Scopus was as follows:

TITLE-ABS-KEY(Pilates OR “Pilates exercise” OR “Pilates training”) AND TITLE-ABS-KEY(“body image” OR “self-esteem” OR “body-esteem” OR “self-perception” OR “social physique anxiety” OR SPAS OR “body dissatisfaction” OR “physical self-concept” OR “physical self-worth”) AND TITLE-ABS-KEY(randomized OR randomised OR trial OR intervention OR “controlled study” OR “controlled trial” OR “quasi-experimental” OR “pre-post”) AND PUBYEAR > 1999 AND PUBYEAR < 2027.

Reference lists of all eligible studies and relevant reviews were manually screened to identify additional records. No language, geographical, or publication date restrictions were applied.

### 2.2. Eligibility Criteria

Eligibility criteria were defined using the PICO framework specified in the PROSPERO protocol (detailed PICO strategy is provided in Supplementary File S2). Studies were eligible if they included healthy individuals (students, adults, older adults, sedentary adults, recreational athletes, obese adolescents/women) or clinical populations (multiple sclerosis, cancer, diabetes, PTSD, anorexia nervosa, juvenile idiopathic arthritis). Elite or professional athletes were excluded because their training load, physiological adaptations, and psychological profiles differ substantially from those of the general population [20], limiting comparability in psychosocial outcomes. Interventions consisted of Pilates-based exercise delivered through mat Pilates, clinical Pilates, tele-Pilates, or home-based programs. While these ‘Pilates-based’ interventions encompass diverse modalities, they are conceptually unified by the core principles of the Joseph Pilates method, such as centering, concentration, and controlled movement. Nonetheless, given this diversity, the present synthesis is framed as an exploratory mapping of the field rather than a definitive assessment of a monolithic intervention. Eligible comparators included usual care, no intervention, waiting-list controls, or non-Pilates exercise. Studies were required to report at least one psychosocial outcome, including mental health indicators (depression, anxiety, psychological distress, self-esteem, self-efficacy, mood, body image) or quality of life (generic or disease-specific). Only randomized controlled trials and quasi-experimental controlled studies were included. Exclusion criteria comprised acute laboratory studies without a comparator, elite athlete samples, studies lacking extractable quantitative data, and non-original research such as reviews, protocols, and conference abstracts.

### 2.3. Selection Process

All records were imported into Mendeley Reference Manager (Version 2.112.0; Elsevier, Amsterdam, The Netherlands) and duplicates were removed. Three independent reviewers screened titles and abstracts, followed by full-text assessment of potentially eligible studies. Disagreements were resolved through discussion and consensus, with a fourth reviewer available for adjudication when necessary. To ensure full reproducibility, the exact dates of data collection and the complete Boolean search strings for each database are provided in the Supplementary File S1. The PRISMA 2020 flow diagram [19] documents the identification of 206 records (PubMed *n* = 37, Scopus *n* = 63, Web of Science *n* = 24, PsycINFO *n* = 28, CINAHL *n* = 33, CENTRAL *n* = 21), removal of 23 duplicates and 19 irrelevant records, screening of 164 titles and abstracts, full-text assessment of 76 reports, exclusion of 44 reports, and inclusion of 32 studies in the final synthesis. Across all included studies, the total sample comprised 1264 participants (Pilates groups: *n* = 632; control groups: *n* = 632).

### 2.4. Data Extraction

Data extraction was performed independently by three reviewers using a standardized form. Extracted information included study characteristics (author, year, country, population), intervention details (duration, delivery mode), comparator type, psychosocial outcomes, and post-intervention means, standard deviations, and sample sizes for experimental and control groups. When multiple psychosocial outcomes were reported, each outcome was extracted as a separate effect size. Additional variables were coded for moderator analyses, including outcome type, population category, age group, delivery mode, and overall risk of bias.

### 2.5. Effect Size Calculation

Effect sizes were calculated as *Hedges*’ *g* [21] using post-intervention means and standard deviations. The pooled standard deviation was computed as follows:

$$\left[SD_{pooled}=\sqrt{\frac{\left(n_{E}-1\right)SD_{E}2+\left(n_{C}-1\right)SD_{C}2}{n_{E}+n_{C}-2}}\right]$$

The small-sample correction factor J was applied:

$$\left[J=1-\frac{3}{4\left(n_{E}+n_{C}\right)-9}\right]$$

The effect size was calculated as follows:

$$\left[g=J\cdot \frac{M_{exp}-M_{ctrl}}{SD_{pooled}}\right]$$

The standard error of g was computed using the following:

$$\left[SE=\sqrt{\frac{n_{E}+n_{C}}{n_{E}n_{C}}+\frac{g^{2}}{2\left(n_{E}+n_{C}-2\right)}}\right]$$

Positive values indicated improvements favoring the Pilates intervention.

### 2.6. Risk of Bias Assessment

Risk of bias was assessed independently by three reviewers using the Cochrane RoB2 tool. Each study was evaluated across domains including randomization, deviations from intended interventions, missing outcome data, outcome measurement, and selective reporting. Studies were categorized as “high risk” or “some concerns”. Discrepancies were resolved through consensus. Risk of bias was included as a moderator in subgroup and meta-regression analyses.

### 2.7. Statistical Analysis

All statistical analyses, including the random-effects models, subgroup analyses, meta-regressions, heterogeneity estimates, sensitivity diagnostics, and publication bias tests, were conducted using JASP statistical software (JASP Team (2026). JASP (Version 0.95.4.0) [Computer software].). The complete dataset used for all analyses is available in Supplementary File S4, and the comprehensive analytical outputs from JASP are provided in Supplementary File S3.

The primary analytical approach was a random-effects model due to expected heterogeneity across populations, intervention formats, and psychosocial outcomes. Between-study variance was estimated using restricted maximum likelihood (REML). Heterogeneity was quantified using *Q*, τ, τ^2^, *I*^2^*,* and 95% prediction intervals. Subgroup analyses were performed for outcome type, age group, delivery mode, population type, and risk of bias. Univariable meta-regressions examined the moderating effects of outcome type, age group, delivery mode, and intervention duration. A multivariable model including outcome type and age group was estimated to assess independent contributions. Sensitivity analyses included leave-one-out diagnostics to identify influential studies and evaluate the stability of pooled effects and heterogeneity estimates. Publication bias was assessed using funnel plot asymmetry and Egger’s regression test, with trim-and-fill procedures planned if asymmetry was detected. In cases where studies reported multiple outcomes for the same participant group (e.g., both quality of life and anxiety scores), all relevant data points were included to maximize the informational yield of this exploratory synthesis. We acknowledge that this approach may impact statistical independence; however, it was deemed necessary to capture the full breadth of psychosocial domains across the heterogeneous evidence base.

## 3. Results

### 3.1. Study Selection Results

**Figure 1:**
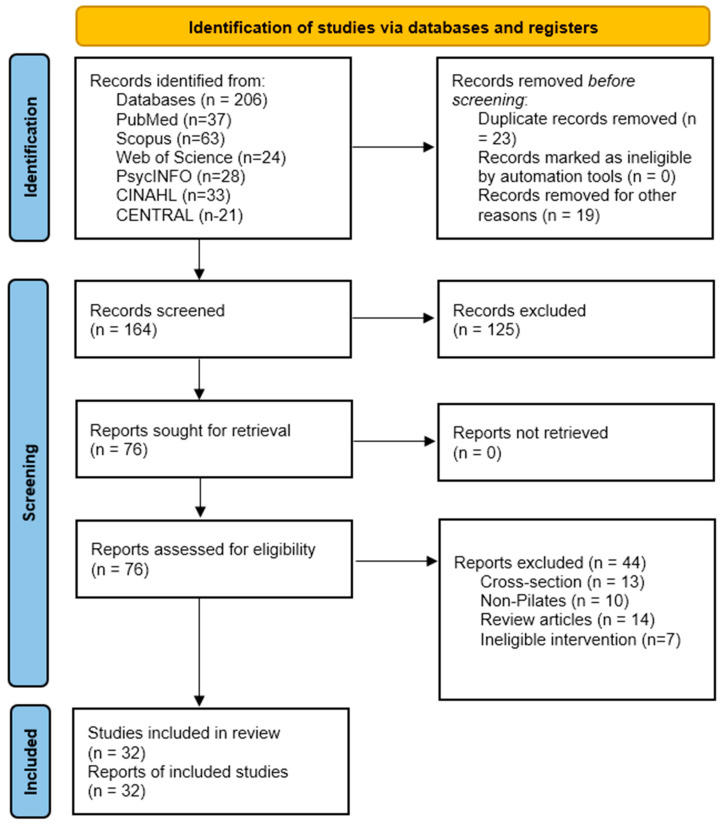
PRISMA flow diagram of the study selection process.

The database search yielded 206 records. After removing 23 duplicates and excluding 19 records for other reasons (e.g., non-journal materials, conference abstracts lacking full texts, or retracted articles), 164 titles and abstracts were screened. Of these, 125 were excluded as irrelevant. Seventy-six full-text articles were assessed for eligibility, and 44 were excluded due to cross-sectional design, non-Pilates interventions, review-type publications, or ineligible intervention characteristics. A total of 32 studies met the inclusion criteria and were included in the review and meta-analysis. The study selection process is presented in the PRISMA 2020 flow diagram (Figure 1).

### 3.2. Study Characteristics

The included studies represented diverse populations, including healthy adults, older adults, adolescents, and clinical groups such as individuals with multiple sclerosis, cancer, diabetes, PTSD, anorexia nervosa, and juvenile idiopathic arthritis. Intervention duration ranged from a single acute session to 52 weeks. Delivery formats included mat-based face-to-face sessions, clinical Pilates, online/tele Pilates, and home-based programs. Psychosocial outcomes were categorized into seven domains: quality of life, self-esteem, well-being, mental health, depression, psychosocial functioning, and body image. The characteristics of the included studies are presented in Table 1.

### 3.3. Overall Effect

The classical unadjusted random-effects model indicated a suggestive but statistically inconclusive overall effect in favor of Pilates (Hedges’ *g* = 0.389, 95% *CI* −0.019 to 0.797, *p* = 0.061). The prediction interval was wide (−1.849 to 2.628), indicating substantial variability in true effects across settings. Heterogeneity was very high (*Q_e_*(31) = 375.34, *p* < 0.001; τ^2^ = 1.164, 95% *CI* 0.700–2.129; *I*^2^ = 91.7%, 95% *CI* 86.9–95.3; *H*^2^ = 12.00, 95% *CI* 7.62–21.12). Table 2 presents the results of the overall random-effects meta-analysis, including the pooled effect size and heterogeneity statistics.

**Figure 2:**
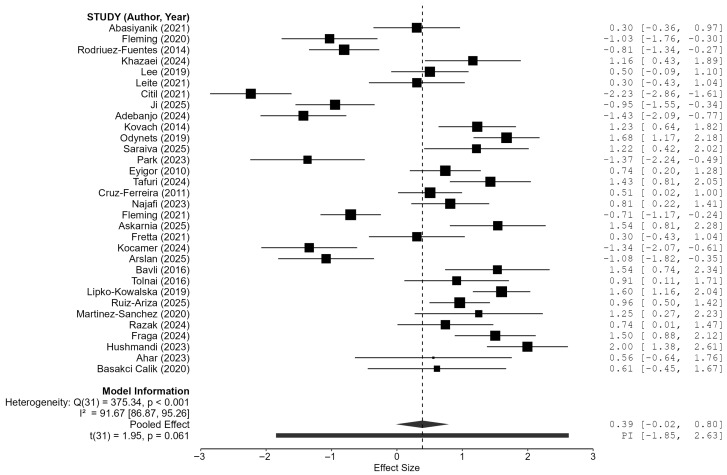
Forest plot of the overall random-effects model including all 32 studies. Squares represent individual study effect sizes (Hedges’ *g*) with 95% confidence intervals; square size reflects study weight. The diamond represents the pooled effect. The vertical solid line represents the null effect (*g* = 0), while the vertical dashed line represents the overall pooled effect size. Full citations for the included studies are provided in the References section and summarized in Table 1 [9,10,11,22,23,24,25,26,27,28,29,30,31,32,33,34,35,36,37,38,39,40,41,42,43,44,45,46,47,48,49,50].

The forest plot (Figure 2) illustrates the individual study effect sizes and the pooled random-effects estimate. Substantial heterogeneity is evident, consistent with the statistical indices reported above.

### 3.4. Subgroup Analyses

#### 3.4.1. Outcome Type

Significant pooled effects were observed for quality of life (*g* = 0.756, *p* = 0.007) and self-esteem (*g* = 0.930, *p* = 0.014). Effects for well-being, mental health, depression, and psychosocial outcomes were non-significant. Subgroup differences were not statistically significant (*Q_m_*(3) = 5.77, *p* = 0.123), partly due to categories with fewer than two studies. Table 3 summarizes the subgroup effects by outcome type, presenting pooled effect sizes, confidence intervals, significance levels, and heterogeneity estimates for each psychosocial domain.

#### 3.4.2. Age Group

Older adults showed a large, significant effect (*g* = 1.115, *p* < 0.001). Other age groups showed non-significant effects. Subgroup differences were significant (*Q_m_*(3) = 10.60, *p* = 0.014).

#### 3.4.3. Delivery Mode

Online/tele-Pilates showed a significant effect (*g* = 0.786, *p* = 0.029), whereas mat-based and clinical Pilates did not. Subgroup differences were significant (*Q_m_*(2) = 10.43, *p* = 0.005).

#### 3.4.4. Population and Risk of Bias

No significant subgroup differences were found for population type (*p* = 0.826) or RoB2 category (*p* = 0.994).

### 3.5. Univariable Meta-Regression Analyses

Outcome type was the only statistically significant moderator (*F_m_*_(6,25)_ = 3.67, *p* = 0.009). Delivery mode (*p* = 0.717), age group (*p* = 0.407), and duration (*p* = 0.745) were not significant moderators. Adjusting for outcome type rendered the pooled effect statistically significant (*g* = 0.393, *p* = 0.023). Residual heterogeneity remained high *(I*^2^ = 87%). Table 4 reports the results of the single-moderator meta-regression models evaluating the influence of study-level variables on effect sizes.

### 3.6. Combined Model

The combined model was statistically significant (F_m_(9,22) = 2.93, *p* = 0.019). Outcome type remained a significant moderator (*p* = 0.012), whereas age group did not (*p* = 0.318). The adjusted pooled effect remained significant (*g* = 0.390, *p* = 0.024). Residual heterogeneity decreased from 92% to 86.7%, indicating partial explanation of variance. Table 5 presents the combined meta-regression model including outcome type and age group as simultaneous moderators.

### 3.7. Sensitivity and Publication Bias Analyses

Sensitivity analyses were conducted to evaluate the robustness of the overall random-effects model and to identify potential influential studies. Casewise diagnostics from the leave-one-out procedure indicated that the pooled effect size remained stable across all iterations. Removing any single study did not meaningfully alter the pooled effect (all changes in *g* < 0.03), the between-study variance (τ ranged from 0.982 to 1.099), or the heterogeneity estimates (*Q_e_* ranged from 302.7 to 375.3). No study produced a shift in the statistical significance of the overall effect. These results confirm that the findings are not driven by any individual study.

Influence diagnostics further supported the absence of influential cases. Standardized residuals ranged from −2.594 to 1.486, with only one value exceeding an absolute magnitude of 2, but without corresponding elevations in Cook’s distance or DFFITS. Cook’s distances were uniformly low (all < 0.20), and DFFITS values were small (all <0.50), indicating that no study exerted disproportionate influence on the model estimates. Covariance ratios were close to 1.00 (0.873–1.068), suggesting that the precision of the model was not substantially affected by the removal of any study. Hat values and study weights were consistent across cases, further confirming the absence of outliers or leverage points.

Taken together, the leave-one-out and influence diagnostics demonstrate that the high heterogeneity observed in the meta-analysis is structural rather than the result of a small number of aberrant studies. The overall effect estimate is therefore considered robust.

Egger’s regression test was computed using the standard linear regression method implemented in JASP. Publication bias was assessed using visual inspection of the funnel plot and Egger’s regression test. The residual funnel plot (Supplementary Figure S1) showed some asymmetry, which is expected given the substantial heterogeneity (τ^2^ = 1.164). Egger’s test, however, was not statistically significant (*p* > 0.05), indicating no evidence of small-study effects. Trim-and-fill analysis did not impute any missing studies, and the adjusted pooled effect remained identical to the original estimate. These results suggest that publication bias is unlikely to have materially influenced the findings.

## 4. Discussion

This meta-analysis synthesized data from 32 randomized and quasi-experimental trials to provide a comprehensive quantification of the psychosocial impact of the Pilates method. While the overall pooled effect indicated a small-to-moderate improvement, the unadjusted model did not reach conventional statistical significance (*p* = 0.061). However, interpreting this unadjusted effect in isolation is methodologically limited due to the extreme structural heterogeneity (*I*^2^ = 91.7%) across the included trials. Crucially, when adjusting for ‘outcome type’ via meta-regression, the model yielded a statistically significant adjusted pooled effect (*g* = 0.393, 95% *CI* 0.058 to 0.728, *p* = 0.023). However, given the extreme variability across populations, interventions, and outcomes, any single summary estimate should be interpreted with considerable caution. Rather than a generalized mental health benefit, our findings confirm that the psychosocial efficacy of Pilates is highly domain-specific and context-dependent. This suggests that outcome type may be a significant moderator of effect variability, although this finding should be considered preliminary and interpreted with caution given the very high heterogeneity and wide prediction intervals observed in the data.

Interpretation of Overall Efficacy and Comparative Literature

The results of this synthesis extend the foundational work of Fleming and Herring [15], whose earlier review was constrained by smaller sample sizes and pre-pandemic data. By incorporating 32 trials from a broader range of countries and populations, the present analysis provides a more robust and contemporary evidence base. However, unlike earlier narrative reviews that suggested improvements in depressive symptoms and anxiety, the present meta-analytic findings do not support statistically significant effects on negative psychological indicators. Mental health outcomes were non-significant, and depression could not be meta-analyzed due to insufficient data. The consistent reduction in depressive symptoms reported in individual studies such as Fleming et al. [37] and Ahar et al. [49] should therefore be interpreted as preliminary and not as pooled evidence. Regarding the potential mechanistic pathways, it is important to note that the following explanations remain speculative, as the aggregated data do not allow for formal mediation testing. Theoretically, Pilates may influence affective states through mechanisms related to self-efficacy, interoceptive awareness, and cognitive-motor engagement; however, these pathways remain hypothetical and cannot be directly inferred from the current results.

A notable pattern emerging from the included studies concerns the effects of Pilates on self-esteem and body image. Research in individuals with obesity [10,40] indicates that the method’s emphasis on controlled movement, proprioception, and non-competitive exercise environments may reduce social physique anxiety and emotional eating while enhancing self-worth. These findings support the notion that the psychological benefits of Pilates are closely tied to attentional focus and body-awareness components [42], which may serve as protective factors against social appearance anxiety [12]. Importantly, the present meta-analysis confirms statistically significant improvements only in positive psychosocial indicators, specifically quality of life and self-esteem, highlighting these domains as the most consistently responsive to Pilates-based interventions. Although these pathways are plausible, the present meta-analysis cannot confirm causality, as most included studies did not measure mediators directly.

Mechanistic Pathways in Clinical Populations: From Chronic Illness to Psychopathology

Subgroup analyses revealed that clinical status shapes the pathways through which Pilates exerts its psychosocial effects, even though clinical status did not emerge as a statistically significant moderator in the meta-regression. In populations with chronic physical illness, such as breast cancer survivors [26,33,38] and individuals with Multiple Sclerosis [9,37], improvements in psychological well-being appear to be accompanied by reductions in pain, fatigue, and functional limitations. This functional-to-psychological pattern is consistent with the broader rehabilitation literature, although the present dataset does not allow formal mediation testing. Findings in children and adolescents with Juvenile Idiopathic Arthritis [50] similarly show that gains in mobility, manual dexterity, and daily functioning co-occur with improvements in biopsychosocial status.

Pilates also appears to be appropriate for populations with specialized psychiatric or metabolic needs. In adolescents with Anorexia Nervosa, Martínez-Sánchez et al. [45] reported reductions in body dissatisfaction and improvements in physical well-being following a 10-week program, suggesting that low-impact, mindful movement may facilitate safer reconnection with the body compared to higher-intensity exercise modalities. Similarly, in older adults with Type 2 Diabetes, Ruiz-Ariza et al. [44] found that 12 weeks of Pilates improved health-related quality of life and nutritional status, indicating potential benefits for self-care behaviors. These findings, while promising, should be interpreted cautiously, as the number of studies per clinical subgroup remains limited.

Delivery Mode: Remote vs. In-Person Applications

One of the key contributions of this meta-analysis is the evaluation of delivery mode. Meta-regression results showed that remote Pilates interventions produce improvements in QoL and mental health comparable to those of in-person instruction in general adult populations [17,32]. This suggests that Pilates may be a scalable intervention suitable for digital health applications, particularly for individuals facing geographical or time constraints. However, evidence from Fraga et al. [47] indicates that in-person delivery may be more effective for older adults, particularly in domains related to psychological and social functioning. These findings imply that while remote formats can address barriers such as limited time availability [42], the interpersonal and supervisory components of in-person sessions may be especially important for populations at risk of social isolation.

Frequency and Sustainability: The “Once-a-Week” Phenomenon

The dataset also highlights the potential efficacy of low-frequency Pilates training. Tolnai et al. [42] demonstrated that even once-weekly sessions can yield improvements in body awareness and affective states among sedentary women. Longer-duration interventions, such as the program examined by Piłsudski and Lipko-Kowalska [43], further support the sustainability of psychological and psychomotor benefits in middle-aged women. These findings suggest that minimal weekly engagement may be sufficient to initiate psychosocial improvements, although the optimal frequency and duration remain unclear due to substantial variability in intervention protocols across studies.

Age and Occupational Contexts as Moderators

Pilates appears to exert age-specific effects. In older adults, improvements in QoL are often linked to enhanced balance confidence and maintenance of independence [6,24,29]. For younger populations, such as university students [8,41], Pilates supports self-esteem development and biomotor improvements. Additionally, evidence from high-stress occupational groups, such as school teachers [11], suggests that Pilates may reduce emotional tension and contribute to workplace well-being. These patterns align with the subgroup findings of the present meta-analysis, although age did not emerge as a statistically significant moderator in the meta-regression.

Stability of Estimates and Interpretive Reliability

Sensitivity analysis confirmed the robustness of the findings. No study exerted disproportionate influence on the pooled effect, and the removal of individual studies did not meaningfully alter estimates of τ or τ^2^. These results indicate that the observed heterogeneity reflects genuine variability in psychosocial responses rather than methodological artifacts. The stability of the effect size across leave-one-out analyses strengthens confidence in the reliability of the conclusions and underscores the structural nature of heterogeneity in mind–body intervention research. Beyond the measured moderators, emerging evidence suggests that instructor-related factors, such as leadership style and class satisfaction, may also influence psychosocial outcomes in Pilates settings. Recent work has reported moderate associations between leadership style, class satisfaction, and psychological well-being, indicating that unmeasured interpersonal and pedagogical variables could contribute to the residual heterogeneity observed in this meta-analysis. Future trials should systematically assess these factors to clarify their role as potential contextual moderators.

Strengths and Limitations

This meta-analysis synthesizes evidence from 32 randomized and quasi-experimental trials across diverse populations and intervention formats, offering a comprehensive and contemporary assessment of the psychosocial effects of Pilates. The use of meta-regression and sensitivity analyses strengthens confidence in the robustness of the findings, and the preregistered protocol, PRISMA 2020 framework, and RoB2 assessment enhance methodological transparency.

A key limitation of this meta-analysis is the very high heterogeneity (*I*^2^ = 91.7%), which reduces the clinical interpretability of the overall pooled effect and underscores the importance of moderator analyses. Several psychosocial domains, such as depression, body image, and psychosocial functioning, could not be meta-analyzed due to insufficient data (<2 studies), limiting conclusions regarding negative psychological indicators. Additionally, differences in study design, intervention characteristics, and measurement approaches significantly influence the interpretation of our findings. The variability in intervention duration, frequency, delivery mode, and the reliance on diverse, self-reported psychometric tools not only constrain comparability across studies but also contribute to the observed risk of bias in several domains. Consequently, the synthesized evidence must be viewed through the lens of these methodological heterogeneities. Despite these limitations, the present synthesis provides robust evidence for positive psychosocial outcomes, particularly quality of life and self-esteem. Future trials should employ more rigorous randomized designs and standardized, validated psychosocial measures to improve comparability. Furthermore, while both acute and chronic interventions were synthesized according to our eligibility criteria, the distinct physiological and psychological mechanisms underlying single-session versus long-term Pilates practice should be carefully distinguished in future primary research. Additionally, our specific subgroup analyses (e.g., in obese populations) primarily reflect female demographics, highlighting a notable absence of controlled Pilates trials targeting male cohorts.

A critical consideration when interpreting the exceptionally high structural heterogeneity (*I*^2^ ≈ 91.7%) and the occasionally contradictory effects observed in this meta-analysis is the inherent tension between statistical consistency and ecological validity. It is necessary to question the very nature of the ‘Pilates’ concept being aggregated. Behind this umbrella label lie multiple distinct practices dependent on parameters that are notoriously difficult to standardize, such as instructor expertise, the choice and sequencing of specific exercises, and the underlying pedagogical intentions. While the statistical aggregation of effects is methodologically justified to detect an overall signal, it simultaneously tends to homogenize deeply heterogeneous clinical realities across diverse target populations and intervention modalities. Therefore, a degree of caution is warranted when generalizing these findings to ‘the’ Pilates method as a uniform, monolithic intervention. To better grasp the specific conditions under which Pilates is most effective, future research must move beyond purely quantitative synthesis and embrace complementary qualitative and contextualized approaches that focus on actual practices, individual experiences, and intervention logic.

Implications for Practice

Pilates appears to be a flexible mind–body intervention capable of supporting a range of psychosocial outcomes across different populations. Remote formats may be suitable for adults with limited access to supervised exercise, whereas in-person instruction may be more appropriate for older adults or individuals at risk of social isolation. Low-frequency programs may be sufficient to initiate improvements, although standardized reporting of training parameters is needed to refine dosage recommendations.

## 5. Conclusions

This exploratory systematic review and meta-analysis provide an updated synthesis of the psychosocial effects of Pilates-based exercise across diverse populations. The overall pooled effect indicated small-to-moderate improvements, although the unadjusted model did not reach statistical significance (*p* = 0.061). Given this non-significant overall effect and the extensive variability across studies, we emphasize that a single summary estimate is insufficient to capture the nuanced effects of Pilates. Instead, the evidence is substantially more consistent for certain psychosocial domains than for a general effect on overall mental health outcomes. Subgroup analyses demonstrated that Pilates yields statistically significant improvements in positive psychosocial indicators, specifically quality of life and self-esteem, whereas effects on well-being, mental health, and other negative psychological indicators were non-significant. Depression and body image outcomes could not be meta-analyzed due to insufficient data, and therefore no conclusions can be drawn regarding these domains.

Meta-regression identified outcome type as the only significant moderator, suggesting that the psychosocial benefits of Pilates depend primarily on the specific domain assessed rather than on participant age, delivery mode, or clinical status. Remote and in-person delivery formats appear comparably effective in general adult populations, although supervised instruction may be more beneficial for older adults or individuals at risk of social isolation. Sensitivity analyses confirmed the robustness of the findings, indicating that no single study disproportionately influenced the results.

Given the substantial heterogeneity and the limited number of studies in several psychosocial domains, further high-quality randomized trials using standardized and validated outcome measures are needed. Overall, the findings support Pilates as a feasible and adaptable mind–body intervention with demonstrated benefits for quality of life and self-esteem, while highlighting the need for more rigorous evidence regarding its effects on negative psychological indicators.

## Figures and Tables

**Table 1:** Characteristics of Included Studies.

Study	Population	Age Group	Delivery Mode	Duration	Outcome	Outcome Type	*N* (Exp)	*N* (Ctrl)
Abasıyanık [9]	Multiple sclerosis	Middle-aged (36–59)	Clinical Pilates	8 weeks	MuSiQoL	QoL	16	12
Fleming [22]	Healthy males	Young adult (18–35)	Mat	Acute	TMD	Well-being	15	15
Rodríguez-Fuentes [23]	Menopause	Middle-aged (36–59)	Mat	8 weeks	MENQOL	QoL	33	20
Khazaei [24]	Elderly males	Older adult (≥60)	Mat	8 weeks	SF-12 MH	Mental health	15	15
Lee [25]	Students	Young adult (18–35)	Mat	8 weeks	RSES	Self-esteem	17	24
Leite [26]	Cancer	Middle-aged (36–59)	Mat	16 weeks	RSES	Self-esteem	13	15
Çitil [27]	PMS	Young adult (18–35)	Mat	12 weeks	PMS Scale	Psychosocial	25	25
Ji [28]	Addiction	Middle-aged (36–59)	Mat	24 weeks	SCL-90-R	Mental health	22	21
Adebanjo [10]	Obese adolescents	Child/Adolescent (<18)	Mat	8 weeks	GHQ-28	Mental health	20	20
Kovách [29]	Elderly	Older adult (≥60)	Mat	24 weeks	WHOQOL	QoL	33	25
Odynets [30]	Cancer	Middle-aged (36–59)	Mat	48 weeks	FACT-B	QoL	40	30
Saraiva [31]	Cancer	Middle-aged (36–59)	Mat	12 weeks	UW-QOL	QoL	13	13
Park [32]	Obesity	Middle-aged (36–59)	Mat	12 weeks	SCL-90-R	Mental health	10	10
Eyigor [33]	Cancer	Middle-aged (36–59)	Mat	8 weeks	QLQ-C30	QoL	26	26
Tafuri [34]	Athletes	Young adult (18–35)	Mat	12 weeks	Self-efficacy	Self-esteem	20	20
Cruz-Ferreira [35]	Healthy women	Middle-aged (36–59)	Mat	24 weeks	SWLS	Well-being	30	32
Najafi [36]	MS	Middle-aged (36–59)	Online	8 weeks	MSQOL-54	QoL	21	22
Fleming [37]	MS	Middle-aged (36–59)	Home-based	8 weeks	QIDS-SR16	Depression	39	36
Askarnia [11]	Teachers	Middle-aged (36–59)	Mat	8 weeks	RSES	Self-esteem	15	15
Fretta [38]	Cancer	Middle-aged (36–59)	Mat	16 weeks	RSES	Self-esteem	13	15
Kocamer [39]	Menopause	Middle-aged (36–59)	Mat	8 weeks	MENQOL	QoL	15	15
Arslan [40]	Obesity	Young adult (18–35)	Mat	8 weeks	SPA	Body image	15	15
Bavli [41]	Students	Young adult (18–35)	Mat	6 weeks	Coopersmith	Self-esteem	10	12
Tolnai [42]	Sedentary adults	Young adult (18–35)	Mat	10 weeks	PANAS-PA	Well-being	13	12
Piłsudski & Lipko-Kowalska [43]	Middle-aged adults	Middle-aged (36–59)	Mat	52 weeks	QoL	QoL	40	40
Ruiz-Ariza [44]	Diabetes	Older adult (≥60)	Mat	12 weeks	EQ-5D	QoL	32	30
Martínez-Sánchez [45]	Anorexia	Child/Adolescent (<18)	Mat	8 weeks	PedsQL	QoL	8	8
Razak [46]	Colorectal cancer	Older adult (≥60)	Online	8 weeks	Global QoL	QoL	15	15
Fraga [47]	Elderly	Older adult (≥60)	Remote	12 weeks	WHOQOL	QoL	13	14
Hushmandi [48]	Obesity	Middle-aged (36–59)	Mat	8 weeks	SWB	Well-being	20	20
Ahar [49]	PTSD	Young adult (18–35)	Mat	8 weeks	Psychological well-being	Well-being	20	20
Basakci Calik [50]	JIA	Child/Adolescent (<18)	Clinical Pilates	8 weeks	PedsQL	QoL	6	9

Note: *N* (Exp) = experimental group sample size; *N* (Ctrl) = control group sample size. Outcome type refers to the psychosocial domain assessed.

**Table 2:** Overall Random-Effects Model.

Statistic	Value
Pooled effect (*g*)	0.389
95% *CI*	−0.019 to 0.797
Prediction interval	−1.849 to 2.628
*p-value*	0.061
*Q*	375.34
*df*	31
*p* (heterogeneity)	<0.001
*I* ^2^	91.67%
τ	1.079
τ^2^	1.164

Note: *g* = Hedges’ g; *CI* = confidence interval; τ^2^ = between-study variance; *I*^2^ = inconsistency index; *Q* = Cochran’s heterogeneity statistic; *df* = degrees of freedom.

**Table 3:** Subgroup Effects by Outcome Type.

Outcome	*g*	95% *CI*	*p*	*I* ^2^
Quality of life	0.756	0.249 to 1.263	0.007	87%
Self-esteem	0.930	0.279 to 1.581	0.014	67%
Well-being	0.597	−0.771 to 1.964	0.293	89%
Mental health	−0.645	−2.587 to 1.298	0.369	91%

Note: Subgroups with fewer than two studies (body image, depression, psychosocial) could not be estimated. *g* = Hedges’ *g*; *I*^2^ = inconsistency index.

**Table 4:** Meta-Regression (Single Moderators).

Moderator	Test	*p*
Outcome_type	*F*_(6,25)_ = 3.667	0.009
Delivery	*F*_(4,27)_ = 0.53	0.717
Age_group	*F*_(3,28)_ = 1.00	0.407
Duration	—	0.745

Note: Tests use Knapp–Hartung adjustment.

**Table 5:** Combined Meta-Regression Model.

Moderator	Test	*p*
Full model	*F*_(9,22)_ = 2.93	0.019
Outcome_type	*F*_(6,22)_ = 3.598	0.012
Age_group	*F*_(3,22)_ = 1.243	0.318
Adjusted pooled effect	*g* = 0.390	0.024
Residual I^2^	86.7%	—

Note: Only outcome type remained a significant moderator when both variables were included.

## Data Availability

Not applicable.

## Supplementary Materials

The following supporting information can be downloaded at: https://www.mdpi.com/article/10.3390/sports14050171/s1, File S1: PRISMA 2020 checklist; File S2: PICO Strategy; File S3: Metanalysis Results; File S4: Pilates Final for JASP; Figure S1: Residual Funnel Plot.

## Abbreviations

The following abbreviations are used in this manuscript:

MuSiQoL	Multiple Sclerosis Quality of Life Questionnaire
TMD	Test of Mood Dimensions
MENQOL	Menopause-Specific Quality of Life Questionnaire
SF-12 MH	Short Form-12 Mental Health subscale
RSES	Rosenberg Self-Esteem Scale
PMS Scale	Premenstrual Syndrome Scale
SCL-90-R	Symptom Checklist-90-Revised
GHQ-28	General Health Questionnaire-28
WHOQOL	World Health Organization Quality of Life Scale
FACT-B	Functional Assessment of Cancer Therapy–Breast
UW-QOL	University of Washington Quality of Life Questionnaire
QLQ-C30	EORTC Quality of Life Questionnaire Core 30
MSQOL-54	Multiple Sclerosis Quality of Life-54
QIDS-SR16	Quick Inventory of Depressive Symptomatology–Self-Report
SPAS	Social Physique Anxiety Scale
Coopersmith	Coopersmith Self-Esteem Inventory
PANAS-PA	Positive Affect subscale of the Positive and Negative Affect Schedule
EQ-5D	EuroQol 5-Dimension Scale
PedsQL	Pediatric Quality of Life Inventory
SWLS	Satisfaction With Life Scale
SWB	Subjective well-being
Global QoL	Global quality of life rating
