# Supplementary material for: Effects of Pilates-Based Exercise on Mental Health, Psychological Well-Being, and Quality of Life: A Systematic Review and Meta-Analysis
Source: Sports (Basel). 2026 Apr 23;14(5):171. doi: 10.3390/sports14050171 (PMC13210596; doi:10.3390/sports14050171)
Supplement: Supplementary file 1 [file sports-14-00171-s001.zip › ‎File S2-PICO Strategy.pdf]

PICO Strategy

Component	Definition (Based on your 32 studies)
**P (Population)**	Clinical populations (MS, Cancer, Diabetes, PTSD, Anorexia, JIA) AND Healthy populations (Students, Elderly, Sedentary, Obese adolescents/women, Athletes).
**I (Intervention)**	Pilates-based exercise: Mat Pilates, Reformer/Clinical Pilates, Tele-Pilates, Home-based (DVD/Online).
**C (Comparator)**	Usual care, no intervention, passive controls (waiting list), or non-Pilates exercise interventions.
**O (Outcome)**	Mental Health (Depression, Anxiety, Self-Esteem, Self-Efficacy, Mood, Body Image) and Quality of Life (Generic or disease-specific scales).
