# Supplementary material for: Effects of Pilates-Based Exercise on Mental Health, Psychological Well-Being, and Quality of Life: A Systematic Review and Meta-Analysis
Source: Sports (Basel). 2026 Apr 23;14(5):171. doi: 10.3390/sports14050171 (PMC13210596; doi:10.3390/sports14050171)
Supplement: Supplementary file 1 [file sports-14-00171-s001.zip › File S3-Metanalysis Results.pdf]

All Article Results

Classical Meta-Analysis

Model Summary

Meta-Analytic Tests

	Test	p
Heterogeneity	$Q_e(31) = 375.34$	< .001
Pooled effect	t(31) = 1.95	.061

Meta-Analytic Estimates

		95% CI		95% PI	
	Estimate	Lower	Upper	Lower	Upper
Pooled effect	0.389	-0.019	0.797	-1.849	2.628
τ	1.079	0.837	1.459		
τ^2	1.164	0.700	2.129		
I^2	91.669	86.871	95.265		
H^2	12.003	7.617	21.117		

Forest Plot

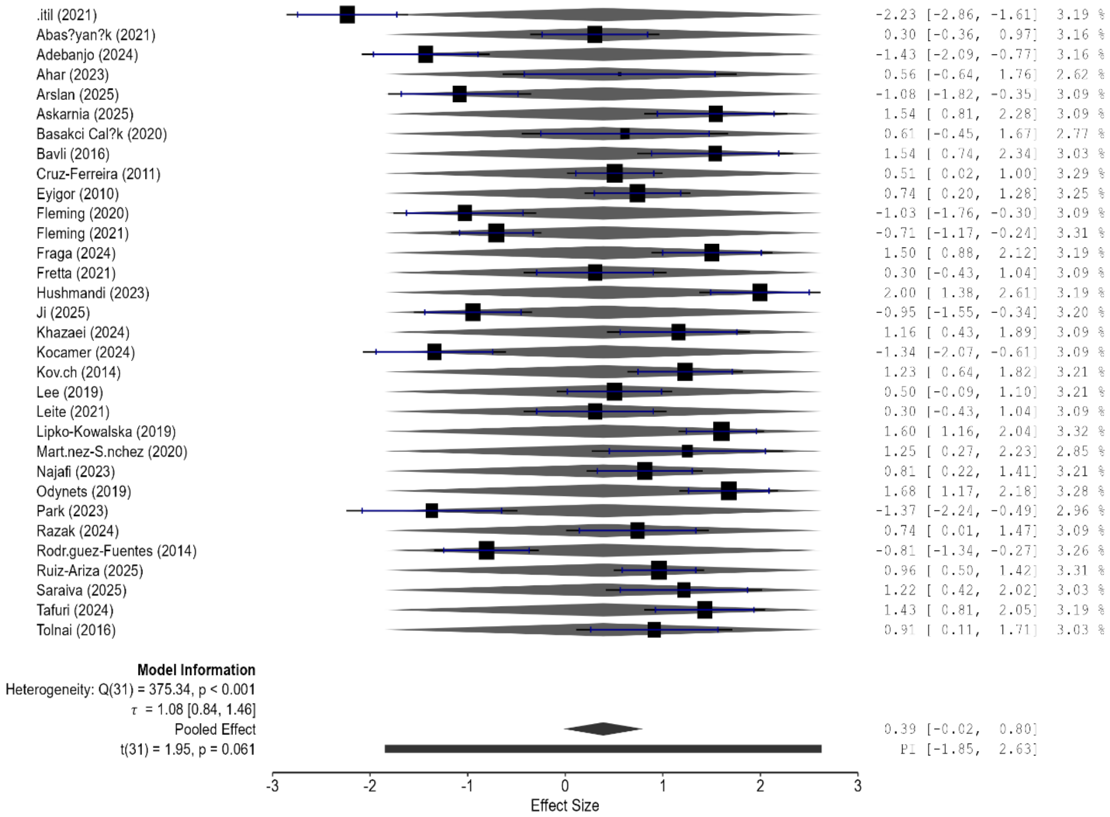

Pilates_Final_for_JASP Outcome_Type Results

Classical Meta-Analysis

Model Summary

Meta-Analytic Tests

	Subgroup	Test	p
Heterogeneity	QoL	$Q_e(13) = 102.89$	< .001
	Well-being	$Q_e(4) = 39.05$	< .001
	Mental health	$Q_e(3) = 32.34$	< .001
	Self-esteem	$Q_e(5) = 14.95$	.011
	Psychosocial		
	Depression		
	Body image		

Meta-Analytic Tests

	Subgroup	Test	p
Pooled effect	QoL	t(13) = 3.22	.007
	Well-being	t(4) = 1.21	.293
	Mental health	t(3) = -1.06	.369
	Self-esteem	t(5) = 3.67	.014
	Psychosocial		
	Depression		
	Body image		
Subgroup differences		$Q_m(3) = 5.77$	.123

Meta-Analytic Estimates

			95% CI		95% PI	
	Subgroup	Estimate	Lower	Upper	Lower	Upper
Pooled effect	QoL	0.756	0.249	1.263	-1.076	2.589
	Well-being	0.597	-0.771	1.964	-2.611	3.804
	Mental health	-0.645	-2.587	1.298	-4.821	3.532
	Self-esteem	0.930	0.279	1.581	-0.517	2.376
	Psychosocial					
	Depression					
	Body image					
τ	QoL	0.815	0.542	1.367		
	Well-being	1.045	0.548	3.099		
	Mental health	1.162	0.582	4.546		
	Self-esteem	0.503	0.144	1.488		

Meta-Analytic Estimates

	Subgroup	Estimate	95% CI		95% PI	
			Lower	Upper	Lower	Upper
	Psychosocial					
	Depression					
	Body image					
τ^2	QoL	0.664	0.294	1.868		
	Well-being	1.092	0.300	9.601		
	Mental health	1.350	0.339	20.663		
	Self-esteem	0.253	0.021	2.215		
	Psychosocial					
	Depression					
	Body image					
I^2	QoL	87.330	75.314	95.094		
	Well-being	89.455	69.969	98.677		
	Mental health	91.231	72.337	99.376		
	Self-esteem	66.880	14.267	94.654		
	Psychosocial					
	Depression					
	Body image					
H^2	QoL	7.893	4.051	20.382		
	Well-being	9.483	3.330	75.578		
	Mental health	11.404	3.615	160.298		
	Self-esteem	3.019	1.166	18.706		

Meta-Analytic Estimates

		95% CI		95% PI	
**Subgroup**	**Estimate**	**Lower**	**Upper**	**Lower**	**Upper**
**Psychosocial**					
**Depression**					
**Body image**					

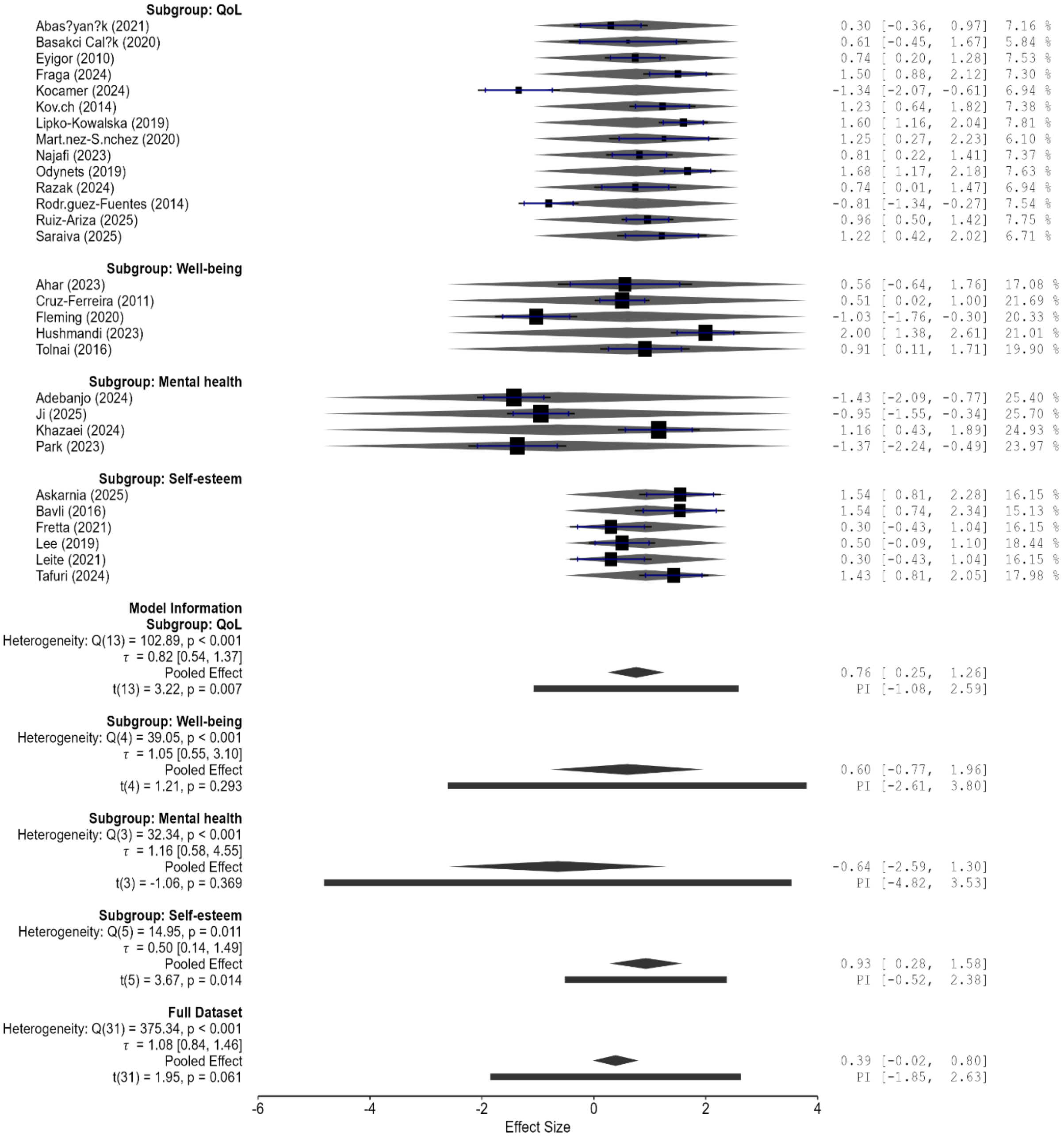

Pilates_Final_for_JASP Age group

Results

Classical Meta-Analysis

Model Summary

Meta-Analytic Tests

	Subgroup	Test	p
**Heterogeneity**	**Middle-aged**	$Q_e(15) = 192.92$	**< .001**
	**Young adult**	$Q_e(7) = 108.54$	**< .001**
	**Older adult**	$Q_e(4) = 3.09$	**.542**
	**Child/Adolescent**	$Q_e(2) = 23.82$	**< .001**
**Pooled effect**	**Middle-aged**	$t(15) = 1.35$	**.197**
	**Young adult**	$t(7) = 0.13$	**.901**
	**Older adult**	$t(4) = 9.25$	**< .001**
	**Child/Adolescent**	$t(2) = 0.13$	**.910**
**Subgroup differences**		$Q_m(3) = 10.60$	**.014**

Meta-Analytic Estimates

			95% CI		95% PI	
	**Subgroup**	**Estimate**	**Lower**	**Upper**	**Lower**	**Upper**
**Pooled effect**	**Middle-aged**	**0.374**	**-0.217**	**0.964**	**-1.954**	**2.702**
	**Young adult**	**0.062**	**-1.085**	**1.210**	**-3.276**	**3.401**
	**Older adult**	**1.115**	**0.780**	**1.450**	**0.780**	**1.450**
	**Child/Adolescent**	**0.105**	**-3.416**	**3.627**	**-6.720**	**6.930**
τ	**Middle-aged**	**1.056**	**0.750**	**1.687**		
	**Young adult**	**1.326**	**0.827**	**2.748**		
	**Older adult**	**0.000**	**0.000**	**0.753**		
	**Child/Adolescent**	**1.359**	**0.605**	**8.791**		
τ^2	**Middle-aged**	**1.116**	**0.562**	**2.845**		
	**Young adult**	**1.758**	**0.685**	**7.554**		
	**Older adult**	**0.000**	**0.000**	**0.567**		

Meta-Analytic Estimates

			95% CI		95% PI	
	**Subgroup**	**Estimate**	**Lower**	**Upper**	**Lower**	**Upper**
	**Child/Adolescent**	**1.846**	**0.367**	**77.289**		
**I²**	**Middle-aged**	**92.350**	**85.878**	**96.853**		
	**Young adult**	**92.822**	**83.435**	**98.232**		
	**Older adult**	**0.000**	**0.000**	**85.353**		
	**Child/Adolescent**	**90.028**	**64.196**	**99.736**		
**H²**	**Middle-aged**	**13.072**	**7.081**	**31.778**		
	**Young adult**	**13.931**	**6.037**	**56.572**		
	**Older adult**	**1.000**	**1.000**	**6.827**		
	**Child/Adolescent**	**10.028**	**2.793**	**379.016**		

Forest Plot

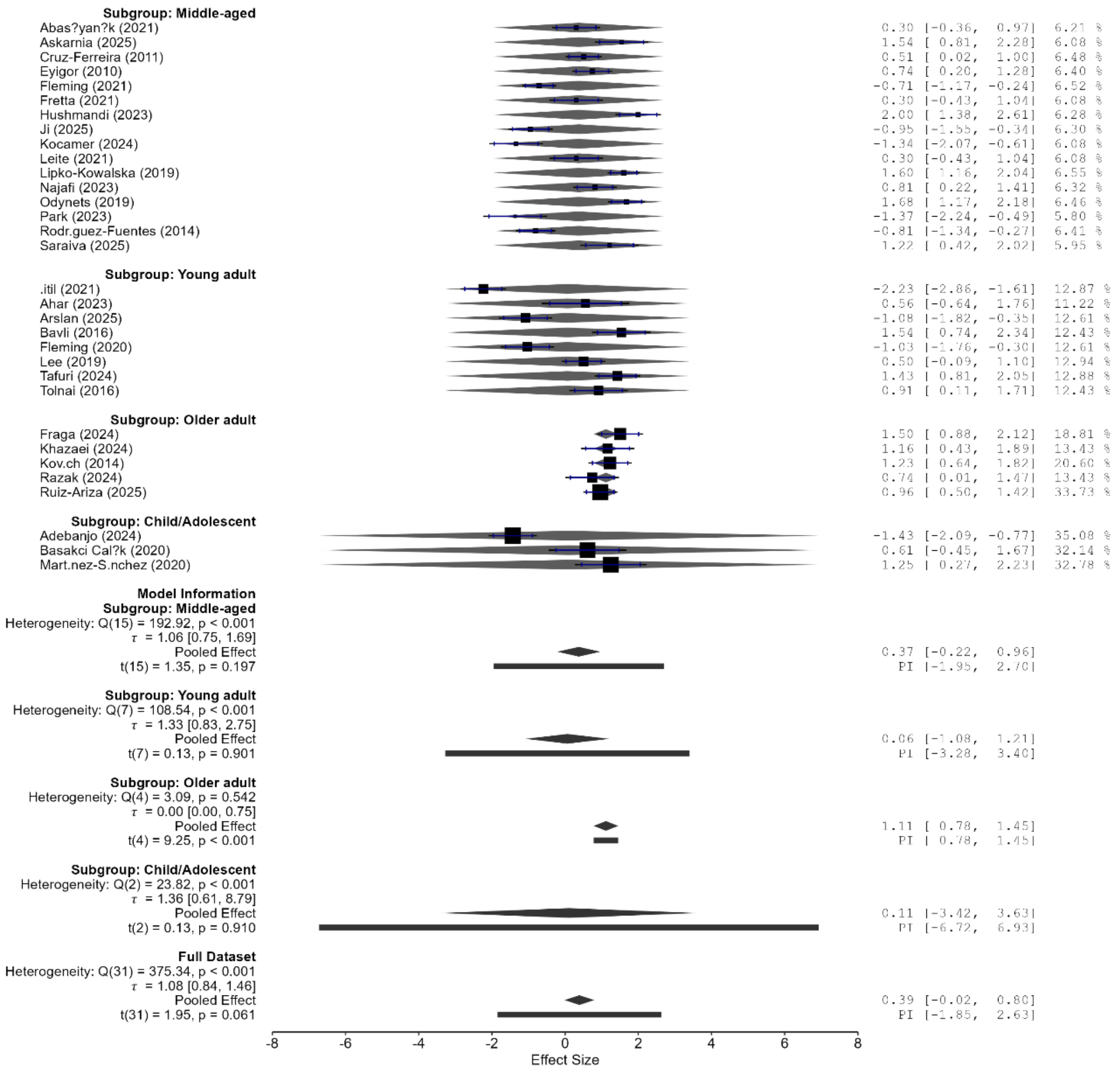

Pilates_Final_for_JASP Delivery

Results

Classical Meta-Analysis

Model Summary

Meta-Analytic Tests

	Subgroup	Test	p
Heterogeneity	Clinical Pilates	$Q_e(1) = 0.24$	.628
	Mat face-to-face	$Q_e(25) = 337.97$	< .001
	Online/Tele	$Q_e(1) = 0.02$	.878
	Home-based		

Meta-Analytic Tests

	Subgroup	Test	p
	Remote		
Pooled effect	Clinical Pilates	t(1) = 2.81	.218
	Mat face-to-face	t(25) = 1.50	.147
	Online/Tele	t(1) = 21.69	.029
	Home-based		
	Remote		
Subgroup differences		$Q_m(2) = 10.43$	.005

Error: The model for subgroup 'Home-based' failed with the following error: Fewer than two estimates.

Error: The model for subgroup 'Remote' failed with the following error: Fewer than two estimates.

Meta-Analytic Estimates

			95% CI		95% PI	
	Subgroup	Estimate	Lower	Upper	Lower	Upper
Pooled effect	Clinical Pilates	0.390	-1.376	2.156	-1.376	2.156
	Mat face-to-face	0.355	-0.134	0.845	-2.088	2.799
	Online/Tele	0.786	0.325	1.246	0.325	1.246
	Home-based					
	Remote					
τ	Clinical Pilates	0.000	0.000	6.958		
	Mat face-to-face	1.162	0.883	1.633		
	Online/Tele	0.000	0.000	1.635		
	Home-based					

Meta-Analytic Estimates

	Subgroup	Estimate	95% CI Lower	95% CI Upper	95% PI Lower	95% PI Upper
	Remote					
τ^2	Clinical Pilates	0.000	0.000	48.409		
	Mat face-to-face	1.351	0.780	2.667		
	Online/Tele	0.000	0.000	2.672		
	Home-based					
	Remote					
I^2	Clinical Pilates	0.000	0.000	99.583		
	Mat face-to-face	92.696	87.997	96.162		
	Online/Tele	0.000	0.000	95.834		
	Home-based					
	Remote					
H^2	Clinical Pilates	1.000	1.000	239.561		
	Mat face-to-face	13.692	8.331	26.058		
	Online/Tele	1.000	1.000	24.004		
	Home-based					
	Remote					

Forest Plot

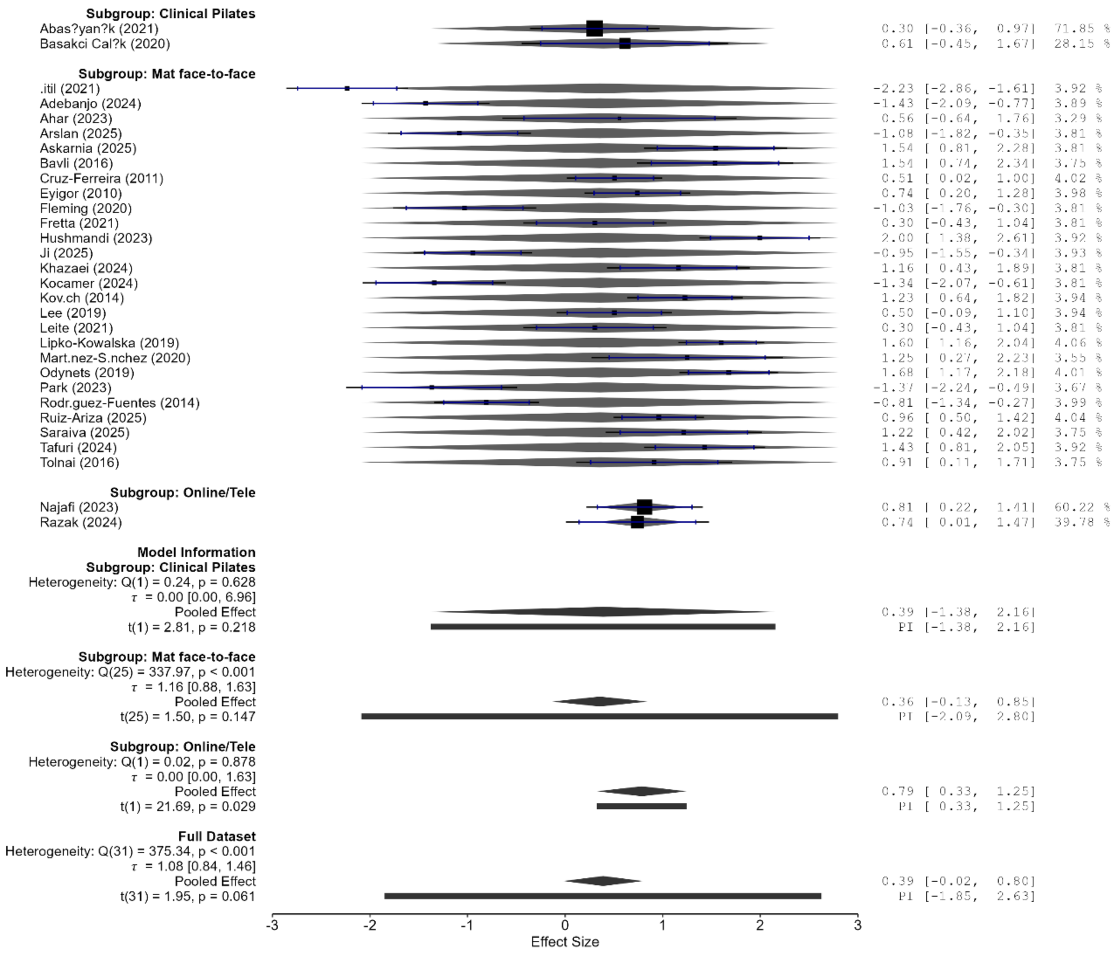

Pilates_Final_for_JASP Population

Results

Classical Meta-Analysis

Model Summary

Meta-Analytic Tests

	Subgroup	Test	p
Heterogeneity	Clinical	$Q_e(17) = 164.44$	< .001
	Healthy	$Q_e(13) = 208.66$	< .001
Pooled effect	Clinical	t(17) = 1.47	.161
	Healthy	t(13) = 1.26	.231
Subgroup differences		$Q_m(1) = 0.05$	.826

Meta-Analytic Estimates

			95% CI		95% PI	
	**Subgroup**	**Estimate**	**Lower**	**Upper**	**Lower**	**Upper**
**Pooled effect**	**Clinical**	**0.348**	**-0.153**	**0.848**	**-1.708**	**2.403**
	**Healthy**	**0.441**	**-0.317**	**1.198**	**-2.401**	**3.282**
**τ**	**Clinical**	**0.945**	**0.665**	**1.456**		
	**Healthy**	**1.268**	**0.889**	**2.086**		
**τ^2**	**Clinical**	**0.893**	**0.442**	**2.120**		
	**Healthy**	**1.607**	**0.791**	**4.352**		
**I^2**	**Clinical**	**89.096**	**80.174**	**95.098**		
	**Healthy**	**93.968**	**88.461**	**97.685**		
**H^2**	**Clinical**	**9.171**	**5.044**	**20.401**		
	**Healthy**	**16.579**	**8.666**	**43.189**		

Forest Plot

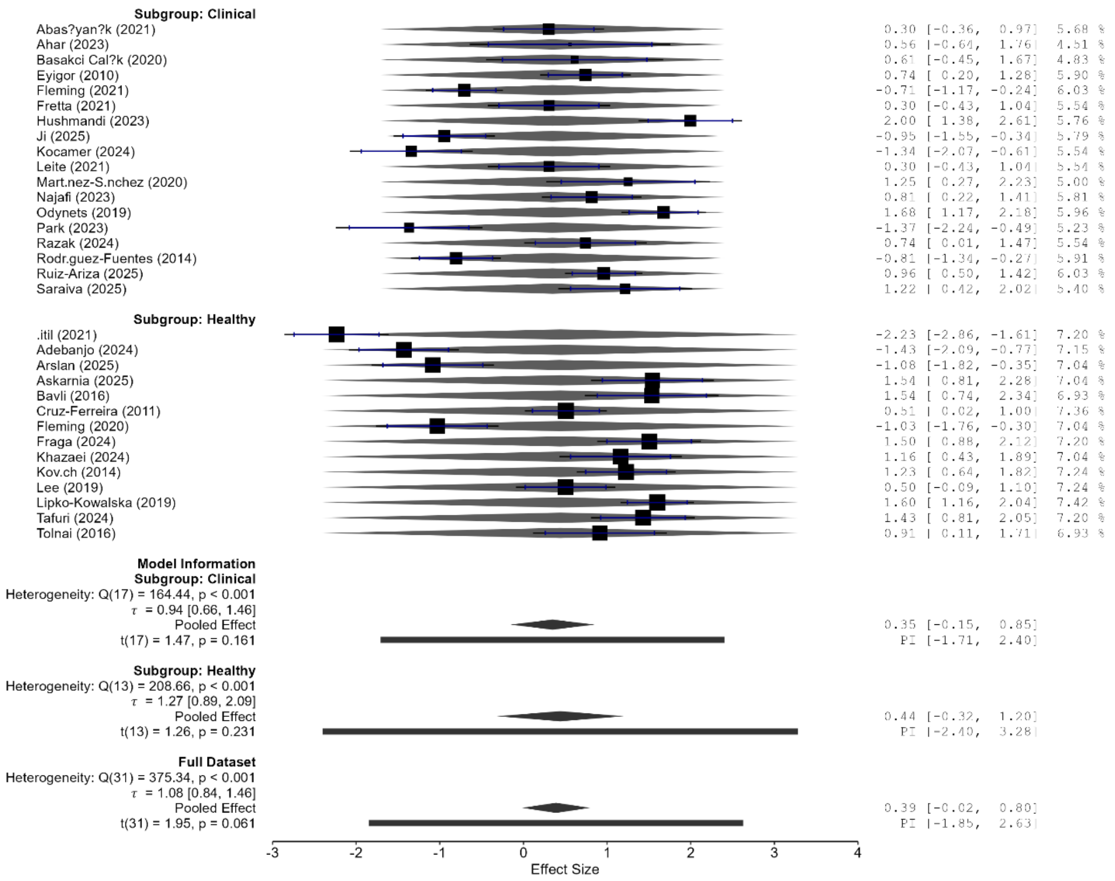

Pilates_Final_for_JASP ROB-2 overall

Results

Classical Meta-Analysis

Model Summary

Meta-Analytic Tests

	Subgroup	Test	p
Heterogeneity	High risk	$Q_e(11) = 113.79$	< .001
	Some concerns	$Q_e(19) = 261.38$	< .001
Pooled effect	High risk	t(11) = 1.35	.205
	Some concerns	t(19) = 1.41	.173
Subgroup differences		$Q_m(1) = 0.00$	.994

Meta-Analytic Estimates

	Subgroup	Estimate	95% CI Lower	95% CI Upper	95% PI Lower	95% PI Upper
**Pooled effect**	**High risk**	**0.391**	**-0.248**	**1.030**	**-1.793**	**2.575**
	**Some concerns**	**0.388**	**-0.186**	**0.962**	**-2.143**	**2.919**
**τ**	**High risk**	**0.949**	**0.631**	**1.676**		
	**Some concerns**	**1.178**	**0.861**	**1.747**		
**τ^2**	**High risk**	**0.900**	**0.398**	**2.811**		
	**Some concerns**	**1.387**	**0.741**	**3.052**		
**I^2**	**High risk**	**90.399**	**80.632**	**96.709**		
	**Some concerns**	**92.387**	**86.641**	**96.391**		
**H^2**	**High risk**	**10.416**	**5.163**	**30.389**		
	**Some concerns**	**13.136**	**7.485**	**27.708**		

Forest Plot

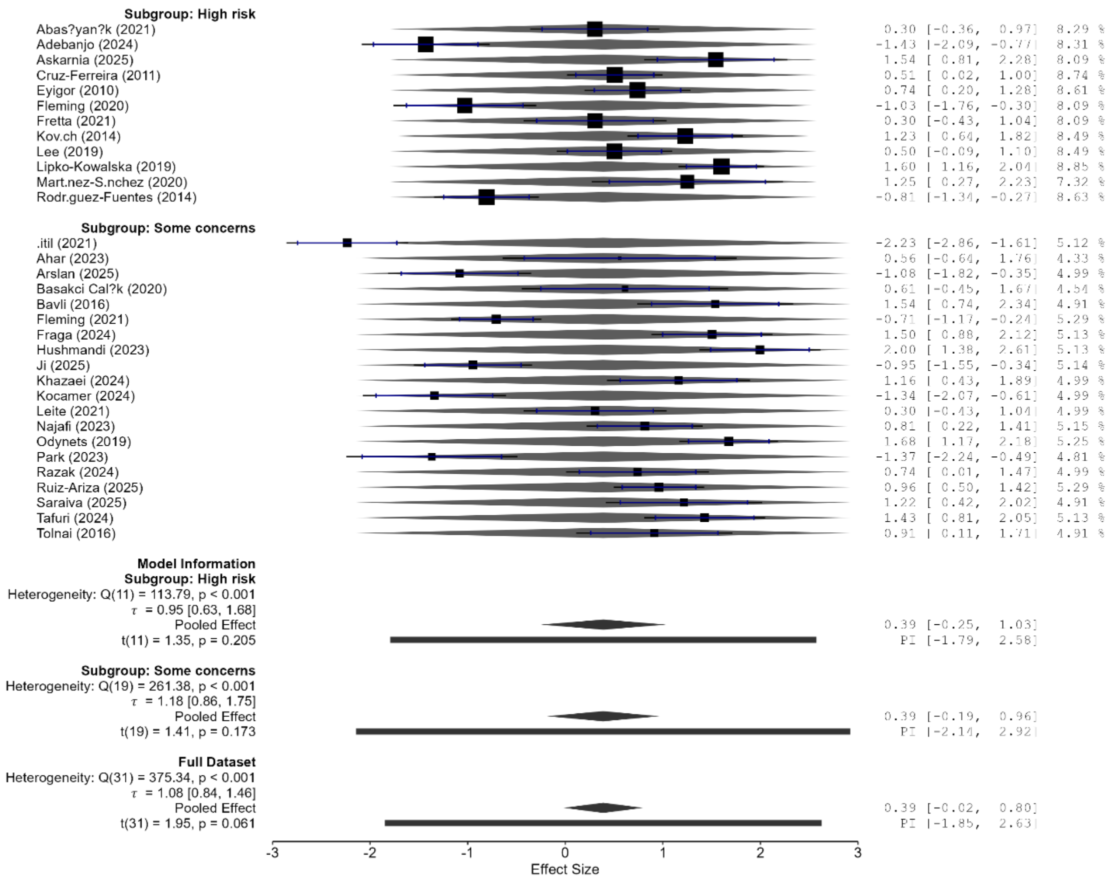

Pilates_Final_for_JASP Metaregration Output type

Results

Classical Meta-Analysis

Model Summary

Meta-Analytic Tests

	Test	p
Residual heterogeneity	$Q_e(25) = 189.24$	< .001
Pooled effect	$t(25) = 2.42$	.023
Moderation	$F_m(6, 25) = 3.67$	.009

Meta-Analytic Estimates

	Estimate	95% CI Lower	95% CI Upper	95% PI Lower	95% PI Upper
**Pooled effect**	**0.393**	**0.058**	**0.728**	**-1.398**	**2.185**
**τ**	**0.854**	**0.631**	**1.218**		
**τ^2**	**0.730**	**0.398**	**1.484**		
**I^2**	**86.994**	**78.479**	**93.147**		
**H^2**	**7.689**	**4.647**	**14.591**		

Note. **The pooled effect size corresponds to the weighted average effect across studies.**

Meta-Regression Summary

Effect Size Meta-Regression Terms Tests

	F	df_1	df_2	p
**Outcome_type**	**3.667**	**6**	**25.00**	**.009**

Note. **Fixed effects tested using Knapp and Hartung adjustment.**

Effect Size Meta-Regression Coefficients

	Estimate	Standard Error	95% CI Lower	95% CI Upper	t	df	p
**Intercept**	**-1.083**	**0.928**	**-2.994**	**0.828**	**-1.167**	**25.00**	**.254**
**Outcome_type (Depression)**	**0.376**	**1.280**	**-2.261**	**3.013**	**0.294**	**25.00**	**.771**
**Outcome_type (Mental health)**	**0.439**	**1.037**	**-1.697**	**2.574**	**0.423**	**25.00**	**.676**

Effect Size Meta-Regression Coefficients

	Estimate	Standard Error	95% CI Lower	95% CI Upper	t	df	p
Outcome_type (Psychosocial)	-1.151	1.298	-3.824	1.522	-0.887	25.00	.384
Outcome_type (QoL)	1.839	0.960	-0.138	3.815	1.916	25.00	.067
Outcome_type (Self-esteem)	2.017	1.001	-0.046	4.079	2.014	25.00	.055
Outcome_type (Well-being)	1.683	1.018	-0.414	3.780	1.653	25.00	.111

Note. **Fixed effects tested using Knapp and Hartung adjustment.**

Forest Plot

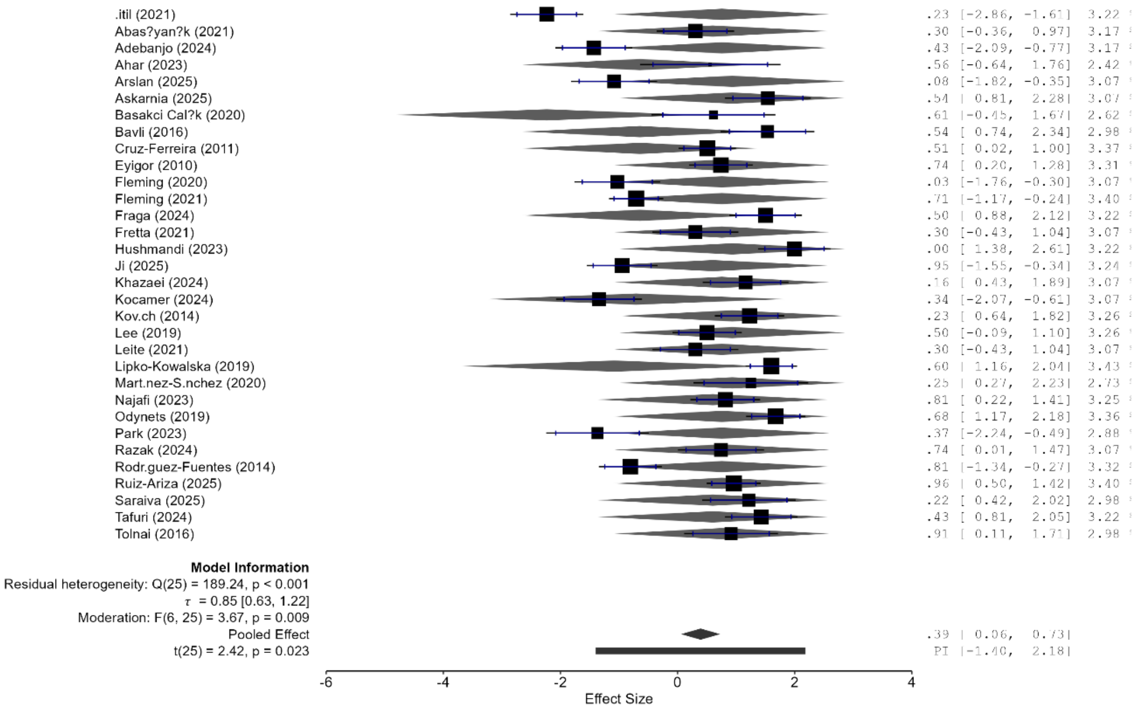

Pilates_Final_for_JASP Meteregration Output plus Age group

Results

Classical Meta-Analysis

Model Summary

Meta-Analytic Tests

	Test	p
Residual heterogeneity	$Q_e(22) = 168.95$	< .001
Pooled effect	t(22) = 2.43	.024
Moderation	$F_m(9, 22) = 2.93$	.019

Meta-Analytic Estimates

		95% CI		95% PI	
	Estimate	Lower	Upper	Lower	Upper
Pooled effect	0.390	0.057	0.723	-1.386	2.166
τ	0.841	0.609	1.231		
τ^2	0.708	0.371	1.515		
I^2	86.656	77.299	93.290		
H^2	7.494	4.405	14.902		

Note. The pooled effect size corresponds to the weighted average effect across studies.

Meta-Regression Summary

Effect Size Meta-Regression Terms Tests

	F	df_1	df_2	p
Age_group	1.243	3	22.00	.318
Outcome_type	3.598	6	22.00	.012

Note. Fixed effects tested using Knapp and Hartung adjustment.

Effect Size Meta-Regression Coefficients

			95% CI				
	Estimate	Standard Error	Lower	Upper	t	df	p
Intercept	-0.737	1.239	-3.308	1.834	-0.595	22.00	.558
Age_group (Middle-aged)	-0.085	0.626	-1.383	1.214	-0.135	22.00	.894
Age_group (Older adult)	0.817	0.687	-0.608	2.242	1.189	22.00	.247
Age_group (Young adult)	-0.346	0.836	-2.080	1.388	-0.414	22.00	.683
Outcome_type (Depression)	0.115	1.378	-2.743	2.972	0.083	22.00	.934
Outcome_type (Mental health)	-0.069	1.185	-2.525	2.388	-0.058	22.00	.954
Outcome_type (Psychosocial)	-1.151	1.279	-3.804	1.502	-0.900	22.00	.378
Outcome_type (QoL)	1.303	1.111	-1.001	3.607	1.173	22.00	.253
Outcome_type (Self-esteem)	1.888	1.025	-0.237	4.012	1.842	22.00	.079
Outcome_type (Well-being)	1.569	1.033	-0.574	3.711	1.519	22.00	.143

Note. Fixed effects tested using Knapp and Hartung adjustment.

Forest Plot

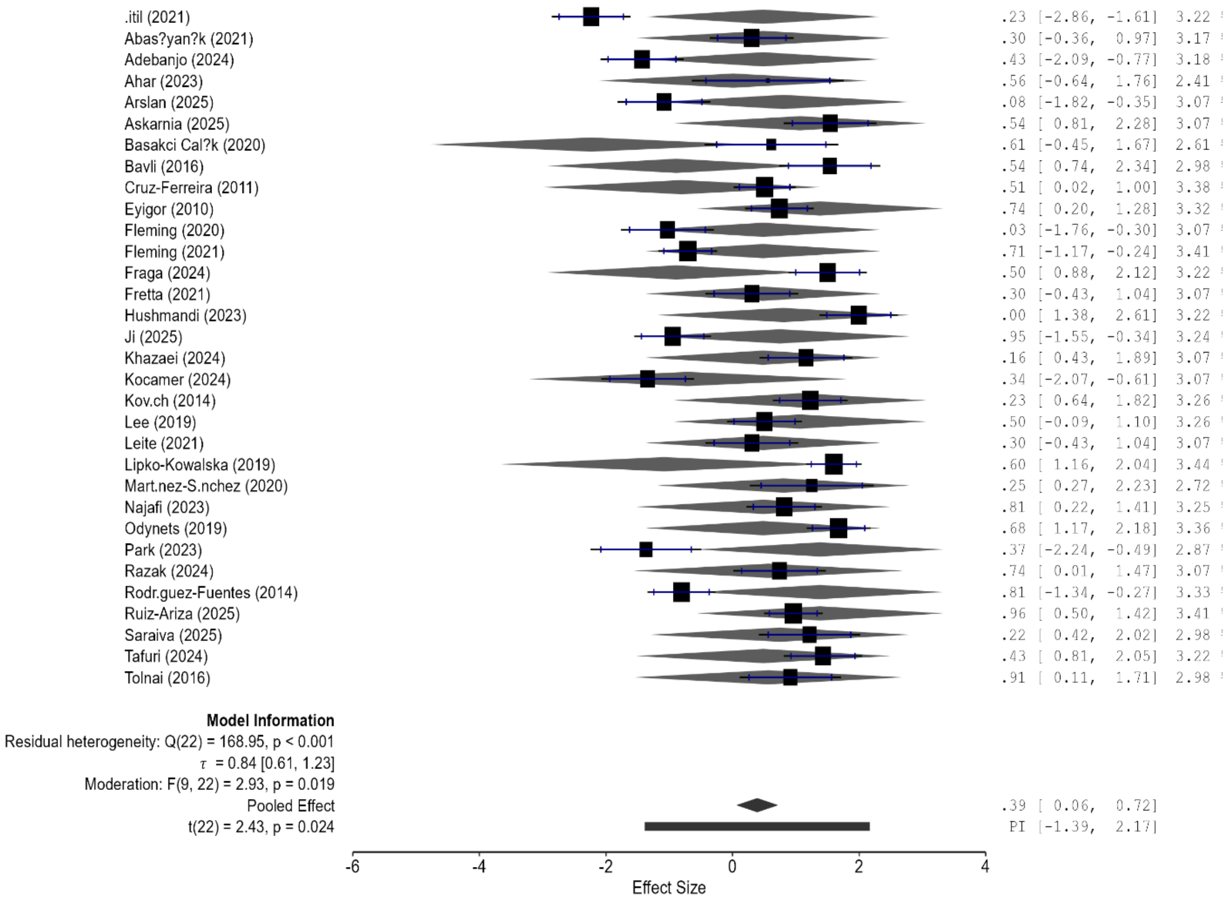

Pilates_Final_for_JASP Metagregration Age Group

Results

Classical Meta-Analysis

Model Summary

Meta-Analytic Tests

	Test	p
**Residual heterogeneity**	**$Q_e(28) = 328.38$**	**< .001**
**Pooled effect**	**t(28) = 1.92**	**.065**
**Moderation**	**$F_m(3, 28) = 1.00$**	**.407**

Meta-Analytic Estimates

		95% CI		95% PI	
	**Estimate**	**Lower**	**Upper**	**Lower**	**Upper**
**Pooled effect**	**0.384**	**-0.026**	**0.794**	**-1.857**	**2.625**
τ	**1.075**	**0.826**	**1.487**		
τ^2	**1.156**	**0.682**	**2.212**		
I^2	**91.618**	**86.563**	**95.435**		
H^2	**11.930**	**7.442**	**21.908**		

Note. **The pooled effect size corresponds to the weighted average effect across studies.**

Meta-Regression Summary

Effect Size Meta-Regression Terms Tests

	F	df_1	df_2	p
**Age_group**	**1.000**	**3**	**28.00**	**.407**

Note. **Fixed effects tested using Knapp and Hartung adjustment.**

Effect Size Meta-Regression Coefficients

			95% CI				
	**Estimate**	**Standard Error**	**Lower**	**Upper**	**t**	**df**	**p**
**Intercept**	**0.084**	**0.675**	**-1.298**	**1.467**	**0.125**	**28.00**	**.901**
**Age_group (Middle-aged)**	**0.289**	**0.731**	**-1.208**	**1.786**	**0.395**	**28.00**	**.696**
**Age_group (Older adult)**	**1.035**	**0.841**	**-0.687**	**2.758**	**1.231**	**28.00**	**.229**
**Age_group (Young adult)**	**-0.027**	**0.787**	**-1.639**	**1.585**	**-0.034**	**28.00**	**.973**

Effect Size Meta-Regression Coefficients

			95% CI				
	Estimate	Standard Error	Lower	Upper	t	df	p

Note. **Fixed effects tested using Knapp and Hartung adjustment.**

Forest Plot

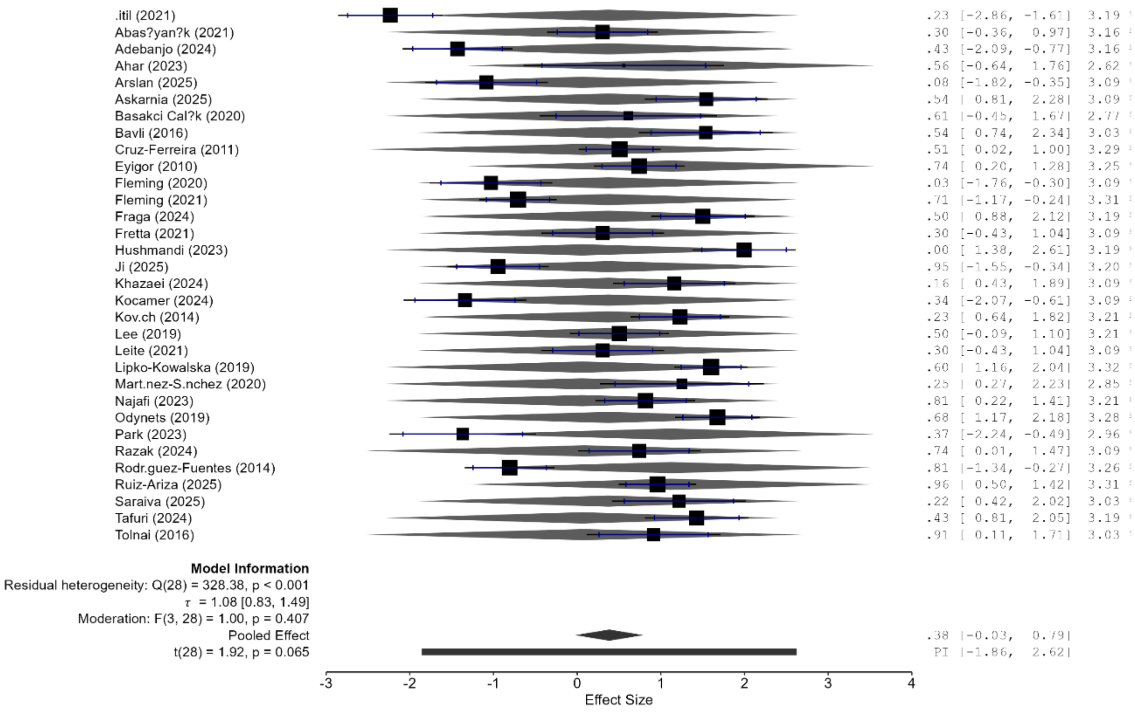

Pilates_Final_for_JASP Metaregration Delivery

Results

Classical Meta-Analysis

Model Summary

Meta-Analytic Tests

	Test	p
Residual heterogeneity	$Q_e(27) = 338.23$	< .001
Pooled effect	t(27) = 1.89	.069
Moderation	$F_m(4, 27) = 0.53$	.717

Meta-Analytic Estimates

		95% CI		95% PI	
	Estimate	Lower	Upper	Lower	Upper
Pooled effect	0.391	-0.033	0.814	-1.939	2.720
τ	1.116	0.853	1.547		
τ^2	1.246	0.728	2.392		
I^2	91.980	87.015	95.654		
H^2	12.468	7.701	23.011		

Note. The pooled effect size corresponds to the weighted average effect across studies.

Meta-Regression Summary

Effect Size Meta-Regression Terms Tests

	F	df_1	df_2	p
Delivery	0.527	4	27.00	.717

Note. Fixed effects tested using Knapp and Hartung adjustment.

Effect Size Meta-Regression Coefficients

			95% CI				
	Estimate	Standard Error	Lower	Upper	t	df	p
Intercept	0.448	0.846	-1.289	2.185	0.529	27.00	.601
Delivery (Home-based)	-1.155	1.417	-4.063	1.753	-0.815	27.00	.422
Delivery (Mat face-to-face)	-0.092	0.877	-1.892	1.707	-0.105	27.00	.917
Delivery (Online/Tele)	0.331	1.180	-2.091	2.752	0.280	27.00	.781
Delivery (Remote)	1.055	1.433	-1.885	3.995	0.736	27.00	.468

Effect Size Meta-Regression Coefficients

	Estimate	Standard Error	95% CI Lower	95% CI Upper	t	df	p

Note. Fixed effects tested using Knapp and Hartung adjustment.

Forest Plot

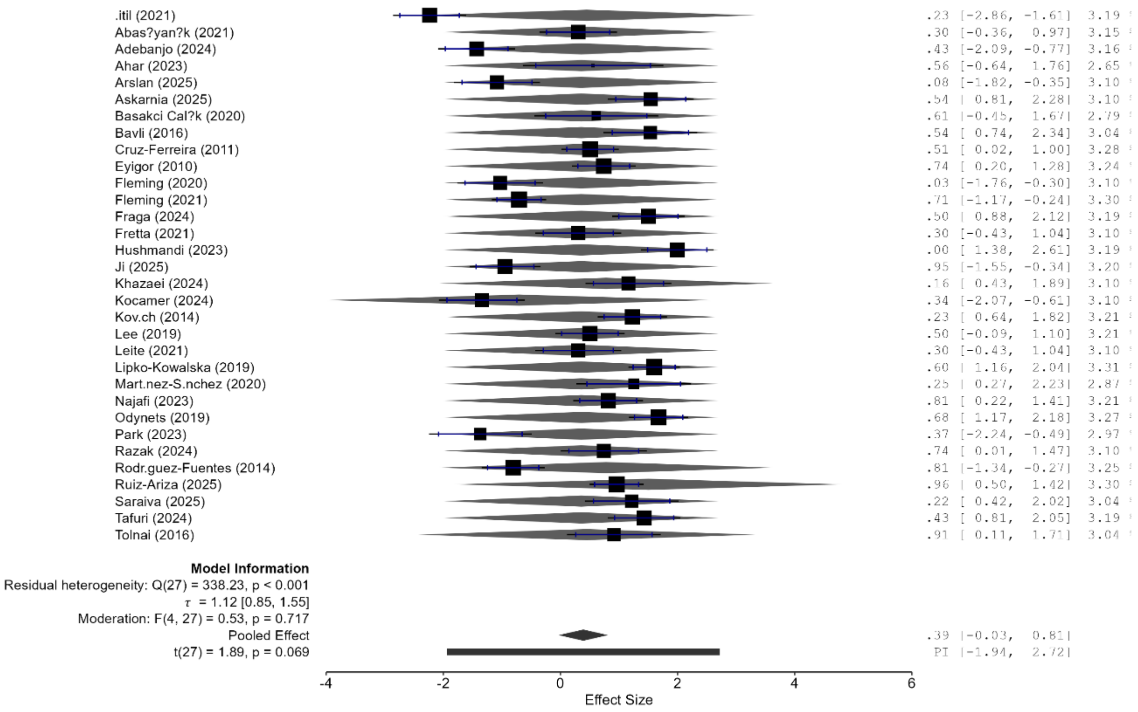

Pilates_Final_for_JASP Metaregration Delivery

Results

Classical Meta-Analysis

Model Summary

Meta-Analytic Tests

	Test	p
**Residual heterogeneity**	**$Q_e(27) = 338.23$**	**< .001**
**Pooled effect**	**$t(27) = 1.89$**	**.069**
**Moderation**	**$F_m(4, 27) = 0.53$**	**.717**

Meta-Analytic Estimates

		95% CI		95% PI	
	**Estimate**	**Lower**	**Upper**	**Lower**	**Upper**
**Pooled effect**	**0.391**	**-0.033**	**0.814**	**-1.939**	**2.720**
τ	**1.116**	**0.853**	**1.547**		
τ^2	**1.246**	**0.728**	**2.392**		
I^2	**91.980**	**87.015**	**95.654**		
H^2	**12.468**	**7.701**	**23.011**		

Note. **The pooled effect size corresponds to the weighted average effect across studies.**

Meta-Regression Summary

Effect Size Meta-Regression Terms Tests

	**F**	df_1	df_2	**p**
**Delivery**	**0.527**	**4**	**27.00**	**.717**

Note. **Fixed effects tested using Knapp and Hartung adjustment.**

Effect Size Meta-Regression Coefficients

			95% CI				
	**Estimate**	**Standard Error**	**Lower**	**Upper**	**t**	**df**	**p**
**Intercept**	**0.448**	**0.846**	**-1.289**	**2.185**	**0.529**	**27.00**	**.601**
**Delivery (Home-based)**	**-1.155**	**1.417**	**-4.063**	**1.753**	**-0.815**	**27.00**	**.422**
**Delivery (Mat face-to-face)**	**-0.092**	**0.877**	**-1.892**	**1.707**	**-0.105**	**27.00**	**.917**
**Delivery (Online/Tele)**	**0.331**	**1.180**	**-2.091**	**2.752**	**0.280**	**27.00**	**.781**

Effect Size Meta-Regression Coefficients

			95% CI				
	Estimate	Standard Error	Lower	Upper	t	df	p
Delivery (Remote)	1.055	1.433	-1.885	3.995	0.736	27.00	.468

Note. Fixed effects tested using Knapp and Hartung adjustment.

Pilates_Final_for_JASP Sensitivity Casewise diagnostics

Results

Classical Meta-Analysis

Model Summary

Meta-Analytic Tests

	Test	p
Heterogeneity	$Q_e(31) = 375.34$	< .001
Pooled effect	t(31) = 1.95	.061

Meta-Analytic Estimates

		95% CI		95% PI	
	Estimate	Lower	Upper	Lower	Upper
Pooled effect	0.389	-0.019	0.797	-1.849	2.628
τ	1.079	0.837	1.459		
τ^2	1.164	0.700	2.129		

Casewise Diagnostics Table

				Leave One Out					Difference in coefficients	
Standardized Residual	DFFITS	Cook's Distance	Covariance ratio	τ	τ^2	Q_e	Hat	Weight	Intercept	Influential
-0.077	-0.014	1.948×10^{-4}	1.067	1.099	1.207	375.2	0.032	3.156	-0.014	
-1.281	-0.229	0.051	1.011	1.067	1.139	359.7	0.031	3.094	-0.229	
-1.101	-0.202	0.041	1.027	1.075	1.156	353.9	0.033	3.256	-0.202	
0.683	0.122	0.015	1.050	1.089	1.187	371.5	0.031	3.094	0.122	
0.103	0.019	3.658×10^{-4}	1.067	1.099	1.207	375.3	0.032	3.214	0.019	
-0.074	-0.013	1.786×10^{-4}	1.066	1.098	1.206	375.2	0.031	3.094	-0.013	
-2.594	-0.473	0.189	0.873	0.982	0.963	302.7	0.032	3.189	-0.472	
-1.224	-0.223	0.049	1.017	1.070	1.144	354.7	0.032	3.203	-0.223	

Casewise Diagnostics Table

Standardized Residual	DFFITS	Cook's Distance	Covariance ratio	Leave One Out			Hat	Weight	Difference in coefficients	
				τ	τ^2	Q_e			Intercept	Influential
-1.692	-0.306	0.088	0.974	1.045	1.092	343.5	0.032	3.161	-0.306	
0.759	0.138	0.019	1.047	1.088	1.183	368.2	0.032	3.214	0.138	
1.192	0.220	0.048	1.020	1.071	1.147	350.9	0.033	3.279	0.220	
0.725	0.128	0.017	1.047	1.088	1.184	371.6	0.030	3.033	0.128	
-1.566	-0.273	0.071	0.985	1.054	1.112	358.9	0.030	2.958	-0.273	
0.316	0.058	0.003	1.064	1.097	1.204	374.1	0.033	3.252	0.058	
0.942	0.171	0.029	1.037	1.081	1.169	365.1	0.032	3.192	0.171	
0.107	0.020	4.032×10^{-4}	1.068	1.099	1.208	375.2	0.033	3.289	0.020	
0.382	0.070	0.005	1.062	1.096	1.201	373.7	0.032	3.211	0.070	

Casewise Diagnostics Table

				Leave One Out					Difference in coefficients	
Standardized Residual	DFFITS	Cook's Distance	Covariance ratio	τ	τ^2	Q_e	Hat	Weight	Intercept	Influential
-1.014	-0.188	0.035	1.033	1.079	1.163	350.6	0.033	3.308	-0.188	
1.031	0.184	0.034	1.030	1.078	1.162	366.3	0.031	3.094	0.184	
-0.074	-0.013	1.786×10^{-4}	1.066	1.098	1.206	375.2	0.031	3.094	-0.013	
-1.582	-0.283	0.076	0.984	1.052	1.107	352.3	0.031	3.094	-0.283	
-1.331	-0.238	0.055	1.007	1.065	1.134	358.5	0.031	3.094	-0.238	
1.014	0.179	0.032	1.030	1.079	1.164	367.9	0.030	3.033	0.179	
0.457	0.081	0.007	1.058	1.094	1.197	373.9	0.030	3.033	0.081	
1.127	0.209	0.043	1.026	1.074	1.153	346.3	0.033	3.323	0.209	
0.521	0.096	0.010	1.059	1.094	1.197	370.0	0.033	3.308	0.096	

Casewise Diagnostics Table

				Leave One Out					Difference in coefficients	
Standardized Residual	DFFITS	Cook's Distance	Covariance ratio	τ	τ^2	Q_e	Hat	Weight	Intercept	Influential
0.733	0.126	0.016	1.045	1.087	1.182	372.6	0.029	2.853	0.126	
0.310	0.055	0.003	1.063	1.096	1.202	374.6	0.031	3.094	0.055	
1.011	0.184	0.034	1.032	1.079	1.164	363.5	0.032	3.192	0.184	
1.486	0.270	0.070	0.994	1.057	1.116	350.1	0.032	3.192	0.270	
0.136	0.022	5.180×10^{-4}	1.060	1.095	1.199	375.3	0.026	2.622	0.022	
0.185	0.031	0.001	1.062	1.096	1.201	375.2	0.028	2.772	0.031	
