# Supplementary figures and images for: Effects of Pilates-Based Exercise on Mental Health, Psychological Well-Being, and Quality of Life: A Systematic Review and Meta-Analysis
Source: Sports (Basel). 2026 Apr 23;14(5):171. doi: 10.3390/sports14050171 (PMC13210596; doi:10.3390/sports14050171)

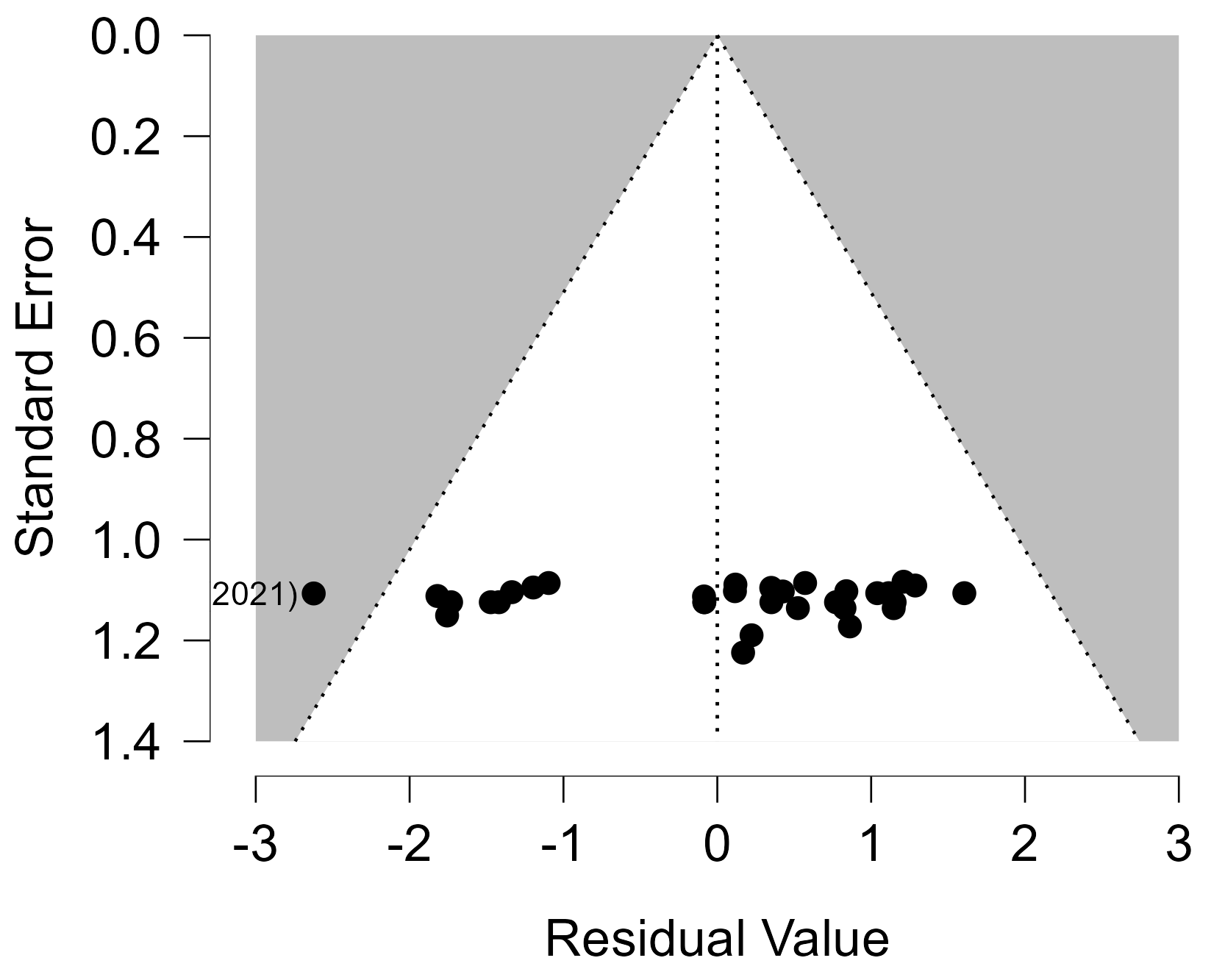

Supplement: Supplementary file 1 [file sports-14-00171-s001.zip › Figure S1.tiff]
